# MiRNA-based expression signatures in differential diagnosis of enchondroma and chondrosarcoma

**DOI:** 10.1016/j.jbo.2026.100761

**Published:** 2026-04-08

**Authors:** Annabell Walter, Kathrin Katenkamp, Nikolaus Gaßler, Thomas Lehmann, Wolfram Weschenfelder, Christian Spiegel, Gunther O. Hofmann, Amer Malouhi, Andreas Hochhaus, Jenny Rinke, Joachim H. Clement, Karin G. Schrenk

**Affiliations:** a Abteilung für Hämatologie und Internistische Onkologie, Klinik für Innere Medizin II, Universitätsklinikum Jena, Jena, Germany; b Mitteldeutsches Krebszentrum Jena/Leipzig, Standort Jena, Jena, Germany; c Institut für Rechtsmedizin, Sektion Pathologie, Universitätsklinikum Jena, Jena, Germany; d Institut für Medizinische Statistik, Informatik und Datenwissenschaften, Universitätsklinikum Jena, Jena, Germany; e Klinik für Unfall-, Hand- und Wiederherstellungschirurgie, Universitätsklinikum Jena, Jena, Germany; f Institut für Diagnostische und Interventionelle Radiologie, Universitätsklinikum Jena, Jena, Germany

**Keywords:** miRNA, miR-138-5p, miR-181a-5p, miR-143-3p, miR-145-5p, Enchondroma, Chondrosarcoma, Biomarkers

## Abstract

**Background:**

The clinical and histological differentiation of benign enchondroma from malignant chondrosarcoma remains a major challenge. Due to their histological similarity, discrimination of enchondroma from low-grade chondrosarcoma G1/ACT particularly complicates accurate diagnosis. MicroRNAs (miRNAs) are promising biomarkers to identify chondrogenic tumors.

**Materials and methods:**

The expression of miR-138-5p, miR-181a-5p, miR-143-3p, and miR-145-5p was assessed by RT-qPCR in tissue samples, as well as in platelets and plasma from healthy donors and patients with enchondroma or chondrosarcoma.

**Results and discussion:**

In chondrogenic tumor tissue compared to adjacent non-tumor tissue the expression of miR-138-5p was significantly increased in enchondroma and low- grade chondrosarcoma G1/ACT, whereas miR-181a-5p expression revealed no significant difference. In contrast, miR-143-3p expression was significantly downregulated in enchondroma and high-grade chondrosarcoma G2 and G3, whereas miR-145-5p exhibited significant downregulation in low-grade chondrosarcoma G1/ACT and high-grade chondrosarcoma G2 relative to the corresponding adjacent non-tumorous tissue. Comparing expression of miR-138-5p, miR-181a-5p, miR-143-3p, and miR-145-5p in enchondroma to chondrosarcoma of different grades, miR-145-5p was significantly decreased in low-grade as well as in high-grade chondrosarcoma and significantly differentiated enchondroma from chondrosarcoma in tissue samples. Analysis in platelets of enchondroma and low-grade chondrosarcoma G1/ACT patients compared to healthy donors revealed no significant change in expression. However, in human plasma significant downregulation of miR-181a-5p and miR-143-3p in enchondroma and low-grade chondrosarcoma G1/ACT patients compared to healthy donors with corresponding ROC curves was demonstrated.

**Conclusion:**

While miR-138-5p and miR-143-3p show potential as tissue-based biomarkers for chondrogenic tumors, miR-145-5p appears to be particularly promising in differentiating enchondroma from chondrosarcoma of all grades. MiR-181a-5p and miR-143-3p exhibit diagnostic value in plasma. However, these findings are exploratory and larger, multicenter studies with independent validation cohorts are essential to confirm their clinical utility to establish robust diagnostic thresholds.

## Highlights

• Distinction between benign enchondroma and malignant chondrosarcoma remains challenging, particularly in borderline lesions.
• MiR-138-5p, miR-143-3p, and miR-145-5p are promising biomarkers for diagnosis of chondrogenic tumors in tissue analysis.
• MiR-145-5p significantly differentiates enchondroma from chondrosarcoma of all grades in tissue samples.
• MiR-181a-5p and miR-143-3p in plasma significantly identify enchondroma and low-grade chondrosarcoma G1/ACT.

## Graphical abstract

**Figure:**
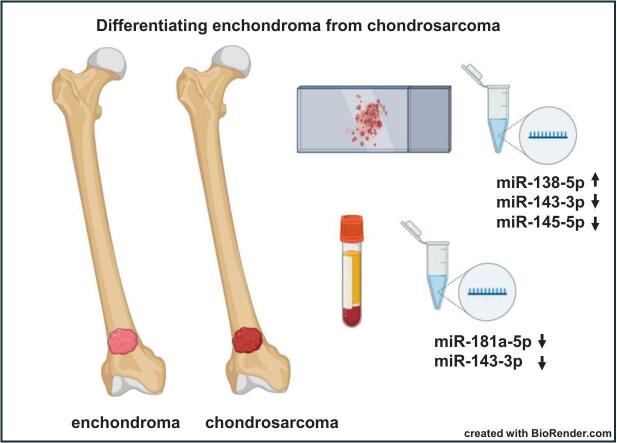

## 1 Introduction

Bone tumors are a rare and heterogenous group of neoplasms with diverse biology and histological features. According to the SEER database, bone and joint cancers represent 0.2% of all new cancer cases in the United States and 0.4% of all cancer-related deaths in 2025. The 5-year relative survival rate for individuals diagnosed with primary bone cancer is estimated at 68.5% for the time period between 2015 and 2021 [1]. The most common types of primary bone cancer are osteosarcoma, chondrosarcoma, and Ewing sarcoma [2], [3], [4]. Chondrogenic tumors include enchondroma and chondrosarcoma. Enchondromas are benign tumors that originate from hyaline cartilage within the medullary cavity [5]. They are the second most common type of benign chondrogenic lesions. Malignant transformation of a solitary enchondroma into a chondrosarcoma is very rare, occurring in about 1% of cases. However, in conditions such as Ollier’s disease or Maffucci’s syndrome, which are characterized by multiple enchondromas, the risk of malignant transformation is significantly higher with 30–35% [6]. Sarcomas are malignant tumors that originate from mesenchymal tissues, such as bone, cartilage, fat, muscle, and connective tissue [7], [8]. They are classified into soft tissue sarcomas and primary bone sarcomas, each with distinct staging systems and treatment strategies [8]. Although sarcomas are rare, they represent over 20% of all pediatric solid cancers compared to less than 1% of adult solid malignancies. Furthermore, soft tissue sarcomas are more common than bone sarcomas [9]. Chondrosarcoma (CS) is the second most common primary malignant bone tumor, accounting for approximately 25% of all biopsied cases, with an incidence of about 3 new cases per 1 million of individuals annually [10], [11]. CS can be classified through histological grading, which is essential for predicting metastatic potential and prognosis. This grading system is crucial for treatment planning and improving patient outcomes [12]. It ranges from grade 1 to 3, determined by the rate of mitosis, necrosis and the amount of dedifferentiation [13], [14]. Grade 1 CS represents low-grade disease, while grades 2 and 3 are high-grade CS carrying a high risk of local recurrence and metastasis [13]. Dependent on localization, low-grade chondrosarcomas are differentiated into low-grade chondrosarcoma G1 with manifestation in the axial skeleton and atypical cartilaginous tumors (ACT) presenting in the extremities. Treatment of chondrosarcoma primarily involves surgical excision, as tumors are often unresponsive to chemotherapy and radiation therapies.

The differentiation between enchondroma and low-grade chondrosarcoma G1/ACT represents a major diagnostic challenge, due to their histological and clinical similarities [15]. Depending on the presence of symptoms such as pain, enchondromas may be managed with a conservative watch & wait approach or intralesional treatments. Chondrosarcoma G1/ACT typically require a more aggressive therapeutic approach with curettage or resection [16]. The distinction of cartilage tumors is currently based on the assessment of imaging and clinical symptoms, as well as possible imaging follow-up examinations [17]. Radiography remains the initial imaging modality for evaluating cartilaginous bone tumors. Enchondromas typically present as well-defined intramedullary lytic lesions with characteristic chondroid matrix mineralization, often described as “rings and arcs” calcifications. These lesions usually show minimal endosteal scalloping, preserved cortical integrity, and absence of periosteal reaction. In contrast, chondrosarcoma G1/ACT may demonstrate more aggressive imaging features, including deep endosteal scalloping, cortical thinning or disruption, bone expansion, and occasionally periosteal reaction. Among these findings, deep endosteal scalloping exceeding two-thirds of the cortical thickness has been identified as one of the most reliable radiographic indicators of chondrosarcoma G1/ACT [18], [19]. Computed tomography (CT) plays an important role in the evaluation of cortical involvement and matrix mineralization. CT allows precise assessment of the extent of endosteal scalloping and detection of subtle cortical breakthrough that may not be clearly visible on radiographs. Several studies have demonstrated that features such as cortical destruction, bone expansion, and periosteal reaction are significantly more common in chondrosarcoma G1/ACT than in enchondroma. CT is therefore particularly useful in cases where radiographic findings are equivocal or when detailed evaluation of cortical integrity is required [18]. Magnetic resonance imaging (MRI) provides superior evaluation of intramedullary tumor extent, adjacent bone marrow changes, and possible extraosseous extension. Both enchondromas and chondrosarcoma G1/ACT typically appear as lobulated lesions with high signal intensity on T2-weighted sequences and low to intermediate signal intensity on T1-weighted sequences due to their high cartilage water content. However, certain MRI findings may favor the diagnosis of chondrosarcoma G1/ACT. These include extensive endosteal scalloping, cortical breach, bone expansion, surrounding bone marrow edema, and soft-tissue edema. The presence of a soft-tissue mass strongly suggests malignant transformation. Nevertheless, enhancement patterns after contrast administration often overlap between benign and low-grade malignant cartilage tumors, limiting the diagnostic specificity of contrast-enhanced MRI [18], [20]. Lesion size and clinical presentation also contribute to the diagnostic assessment. Enchondromas in long bones are usually smaller than 5 cm and frequently discovered incidentally in asymptomatic patients. In contrast, chondrosarcoma G1/ACT lesions tend to be larger and may be associated with localized pain unrelated to fracture. Pain attributable to the lesion has been reported as one of the most significant clinical predictors of chondrosarcoma G1/ACT. In addition, interval growth of a cartilaginous lesion in a skeletally mature patient should raise suspicion for malignant transformation. Established decision algorithms such as the RAS criteria and the Birmingham Atypical Cartilage Tumour Imaging Protocol (BACTIP) integrate radiological, morphologic, and clinical variables to refine risk stratification, albeit with limited accuracy in indeterminate lesions [21], [22]. The BACTIP criteria are used for highly differentiated cartilage tumors in common locations around the knee joint and in the proximal humerus. These criteria do not recommend follow-up for small, inconspicuous lesions (known as IA lesions), but recommend MRI follow-up after one or three years for other lesions without clear signs of malignancy. Imaging characterisation of highly suspicious lesions follows the RAS criteria [21], [22]. Radiomics-based models have achieved high discriminatory performance, yet moderate interobserver variability and biopsy undergrading remain notable challenges, particularly in medium-sized (4–7  cm) lesions exhibiting intermediate features. Consequently, neither conventional imaging nor histopathology alone provides definitive classification. Integration of molecular biomarkers into radiologic scoring frameworks may improve diagnostic precision and objectivity. Developing integrated clinical, radiological, and molecular algorithms may help reduce overtreatment of benign lesions and prevent delays in identifying malignant transformation.

Various potential biomarkers for the differentiation of chondrogenic tumors have been proposed in the last decades. Although many of these markers show promising results, no reliable and standardized markers are available for clinical use [23]. Prominent examples for genetic markers of chondrogenic tumors include isocitrate dehydrogenase (IDH) 1 and 2 mutations. Up to 50% of chondrogenic tumors harbour these mutations [24], [25]. Although IDH mutations are important genetic markers of chondrosarcoma, they are found in benign chondrogenic and other malignant tumors, respectively. This limits their effectiveness as a diagnostic tool for differentiation of chondrogenic tumors [26].

Promising candidates in the differentiation of chondrogenic tumors are microRNAs (miRNAs), non-coding single-stranded RNAs, that are approximately 23 nucleotides in length [27]. They have an important role in regulating gene expression, which occurs mainly at the post-transcriptional level [28], [29]. Since their discovery in 1993, miRNAs have been recognized as key regulators of cellular processes and disease mechanisms [28], [30], [31]. MiRNAs exert their regulatory function through various mechanisms of post-transcriptional gene regulation, causing translational repression and affecting mRNA stability by deadenylation and decapping or degradation of the targeted mRNA [32], [33]. One single miRNA can target multiple mRNAs, while one mRNA may contain several binding sites for different miRNAs. This enables the regulation of a wide range of biological processes including cancer [32], [34].

In chondrogenic tumors Zhang et al., 2016 [35] analyzed miR-138-5p and miR-181a-5p for the differentiation between enchondroma and low-grade chondrosarcoma. Their expression was increased in human formalin-fixed paraffin-embedded (FFPE) tissues of low-grade chondrosarcoma patients compared to enchondroma patients. Urdinez et al., 2020 demonstrated miR-143-3p and miR-145-5p to be downregulated in FFPE tissues and plasma from chondrosarcoma patients compared to enchondroma patients [36].

In this study we sought to investigate the potential of miR-138-5p, miR-181a-5p, miR-143-3p, and miR-145-5p as biomarkers for differentiating chondrogenic tumors in tissue samples. Moreover, we analyzed their use in liquid biopsies for the particular challenging discrimination between enchondroma and low-grade chondrosarcoma in platelets and plasma.

## 2 Materials and methods

### 2.1 Patients and samples

All human tissue samples were collected between 2005 and 2024 at the University Hospital Jena. Formalin-fixed, paraffin-embedded (FFPE) non-necrotic tissues were employed for analysis. The study was approved by the Ethics Committee of the Friedrich-Schiller-Universität Jena. 43 patients were included. Informed consent for the use of tissue sections was obtained from 24 patients or their relatives. 19 patients could not be reached because they had moved or died and no family contact was available. Clinical characteristics of the patients were age, gender, disease stage, grade, time of first presentation, affected organ, and time to metastasis (Table 1). As indicated in Table 1, in several tissue samples no adjacent non-tumor tissue was present. For miRNA expression analysis in human blood samples, 12 healthy individuals and 11 patients with enchondroma or low-grade chondrosarcoma G1/ACT were included for miRNA analysis in platelets or plasma (Table 2). Informed consent was obtained from all healthy participants and patients donating blood samples.

**Table 1:** Clinical data of 16 enchondroma and 27 chondrosarcoma patients G1/ACT, G2, and G3 with analysis of FFPE tissue samples.

Variable		Enchondroma	Chondrosarcoma		
			G1/ACT	G2	G3
Number		16	11	10	6
Age (years)	Median	44	49	56.5	69.5
	Range	(8 - 65)	(8 - 74)	(26 - 85)	(53 - 81)
Gender	Male	9	3	9	4
	Female	7	8	1	2
Tissue type	Tumor	14	10	10	6
	Adjacent	6	8	7	5
Tumor site	Femur	4	4	3	4
	Tibia	1	1	1	1
	Humerus	2	1	1	0
	Pelvis	0	1	2	0
	Phalanx	8	3	1	1
	Other	1	1	2	0
Tumor size	T1		6	3	2
	T2		4	6	1
	T3		0	0	2
	T4		0	1	1
	n/a		1	0	0
Stage of disease	Localized		10	8	3
	Metastatic		1	2	3

**Table 2:** Clinical data from blood donors, including healthy controls (n = 12), enchondroma patients (n = 6) and low-grade chondrosarcoma G1/ACT patients (n = 5) for platelet and plasma cohort.

**Variable**		**Healthy controls**	**Enchondroma**	**Chondrosarcoma G1/ACT**
**Number**		12	6	5
**Age (years)**	Median	27	36	53
	(Range)	(20–66)	(32–64)	(39–64)
**Gender**	Male	5	1	2
	Female	7	5	3
**Tumor site**	Femur		4	1
	Fibula		0	1
	Scapula		0	1
	Pelvis		0	1
	Phalanx		2	0
	Thorax (ribs)		0	1
**Tumor size**	T1			3
	T2			1
	T3			0
	T4			0
	n/a			1
**Stage of disease**	Localized			5
	Metastatic			0

### 2.2 Assessment of tumor and adjacent non-tumor tissue

Adjacent non-tumor tissue was defined as cartilage tissue located adjacent to the tumor that showed no histological signs of malignancy. For each FFPE block, 1.5 µm sections were cut and stained with hematoxylin and eosin (H&E) according to standard protocols. All slides were reviewed by an experienced pathologist, specialized in sarcoma diagnostics. Regions classified as adjacent non-tumor tissue had to display preserved cartilage architecture and absence of tumor-associated features such as increased cellularity, nuclear atypia or binucleation. To avoid contamination with tumor or reactive areas, only clearly separable regions without evidence of tumor infiltration were selected for analysis. The annotated H&E slides served as the basis for tissue sampling and subsequent miRNA isolation, ensuring separation of tumor and adjacent non-tumor compartments.

### 2.3 Isolation of RNA from FFPE tissue sections

The purification of total RNA, including miRNAs, from formalin-fixed, paraffin-embedded tissue sections was performed using the miRNeasy FFPE-Kit (QIAGEN, Hilden, Germany) following the manufacturer's protocol. Hematoxylin and eosin staining was used to enable discrimination between tumor and adjacent non-tumor tissue. After deparaffinization and drying, adjacent non-tumor and tumor tissues were precisely removed from the slides using a sterile scalpel and transferred into nuclease-free tubes. The concentration and purity of the isolated RNA was assessed using a Nanodrop 2000 spectrophotometer. Subsequently, the extracted RNA was either stored at −80°C for future experimentation or directly utilized for reverse transcription.

### 2.4 cDNA synthesis from FFPE tissue sections

For cDNA synthesis and subsequent quantitative reverse transcription polymerase chain reaction (RT‑qPCR) analysis, the All-in-one™ kit 2.0 (GeneCopoeia, Rockville, MD, USA) was utilized. A maximum of 300 ng total RNA was used for each sample. cDNA synthesis was completed using a TRIO thermocycler (Biometra, Göttingen, Germany). The first step involved incubation at 37°C for 60 min to allow activation of the enzymes in the mixture and facilitate the reverse transcription process. Subsequently, the enzymes were inactivated by heating up the reaction mix to 85°C for 5 min. The resulting cDNA was either used immediately or stored at −20°C until future use.

### 2.5 RT‑qPCR from FFPE tissue sections

Following cDNA synthesis, RT-qPCR was performed using the All-in-one™ kit 2.0 (GeneCopoeia). *SNORD49A* and *RNU6-2* were selected as reference RNAs for normalization in RT-qPCR to ensure that variations in RNA quantity did not influence the results. All-in-One™ miRNA qPCR primers were used as forward primers (Suppl. Table 1). The RT-qPCR was conducted using the Rotor-Gene system (QIAGEN). Reactions were performed in duplicates, and negative controls were included, consisting of reactions with sterile ddH_2_O. Melting curve analysis verified the specificity of the PCR products, ensuring that the amplification was accurate and that no primer-dimers or non-specific products were present.

### 2.6 Preparation of human platelets

Human blood (10 ml) was collected into sterile citrate-containing tubes. The citrate tubes were centrifuged at room temperature for 30 min at 150 g without using a brake. The upper layer (platelet-rich plasma, PRP) was transferred into a new 15 ml Falcon tube. A second centrifugation with 300 g for 10 min at room temperature was performed. The supernatant was removed and the pellet lysed in 700 µl QIAzol (QIAGEN). The homogenized cell lysates were either immediately utilized for RNA isolation or stored at −80°C for future use.

### 2.7 Isolation of microRNAs from human platelets

Frozen homogenized lysates were thawed in a 37°C water bath (GFL 1002) until fully dissolved. The miRNeasy Micro Kit (QIAGEN) was utilized for the purification of miRNAs from human platelets. The procedure was performed in accordance with the manufacturer's instructions.

### 2.8 cDNA synthesis from human platelets

After isolation of the miRNA-enriched fraction from platelets cDNA synthesis and RT-qPCR were performed. The All-in-one™ kit 2.0 (GeneCopoeia) was used to analyze miRNA expression in platelets, following the same approach as for patient tissues. A fixed volume of 5 µl of isolated RNA was used for cDNA synthesis. cDNA synthesis was completed using a TRIO thermocycler (Biometra). The resulting cDNA was either immediately used for RT-qPCR or stored at −20°C for future use.

### 2.9 RT-qPCR from human platelets

RT-qPCR was performed for miRNA expression analysis in platelets using the All-in-one™ kit 2.0 (GeneCopoeia). For normalization miR-16-5p, miR-423-3p, and miR-let-7b-5p were used as reference miRNAs to account for variations in RNA input (Suppl. Table 1). Samples were analyzed in duplicates, and negative controls were included by replacing the diluted cDNA with sterile ddH_2_O. To confirm the specificity of the PCR products, a melting curve analysis was carried out, ensuring the absence of primer-dimers or unintended amplification products.

### 2.10 Preparation of human plasma

Human blood (10 ml) was collected in EDTA-containing tubes and blood was processed within one hour after collection. Blood samples were centrifuged in primary blood collection tubes at 1900 g and 4°C for 10 min using a swinging bucket rotor. The upper phase was transferred into a new tube without disturbing the intermediate buffy coat layer and a high-speed centrifugation step was conducted for 10 min at 16.000 g and 4°C. For extended storage, plasma aliquots were frozen at −80°C.

### 2.11 RNA isolation from human plasma

Plasma was either used immediately after preparation or thawed at room temperature prior to nucleic acid purification. The miRNeasy Serum/Plasma Advanced Kit (QIAGEN) was utilized according to the manufacturer's protocol. Control for the quality of RNA isolation and identification of experimental outliers was performed with the RNA Spike-in Kit (QIAGEN). The UniSp2, UniSp4, and UniSp5 RNA Spike-in Mix was prepared according to the kit's manual. The isolated RNA was either immediately used for downstream cDNA synthesis or stored at −80°C until further analysis.

### 2.12 cDNA synthesis from human plasma

For cDNA synthesis of miRNAs isolated from human plasma, the miRCURY LNA RT Kit (QIAGEN) was utilized. As a control for the quality of cDNA synthesis, the UniSp6 RNA spike-in template was employed, which is provided with the miRCURY LNA RT Kit. After PCR, RNA spike-in levels were compared and potential outliers were identified and excluded from further data analysis. After preparing the reaction mix, cDNA synthesis was carried out using a TRIO thermocycler (Biometra). The reverse transcription reaction was performed at 42°C for 60 min followed by heat inactivation of the reverse transcriptase at 95°C for 5 min.

### 2.13 RT-qPCR from human plasma

RT-qPCR was performed for miRNA expression analysis in human plasma using the miRCURY LNA SYBR® Green PCR Kit (QIAGEN). As reference miRNAs for normalization in RT-qPCR miR-30e-5p, miR-93-5p, and miR-16-5p were selected. Spike-ins UniSp2 and UniSp6 were quantified for control of RNA isolation and cDNA synthesis (Suppl. Table 2). The primers of the miRCURY LNA miRNA PCR Assays (QIAGEN) were used.

### 2.14 Exclusion criteria

Samples derived from tissue, platelets, and plasma with RNA yields below 30  ng/µL or purity values not meeting established quality thresholds (A260/280 < 2.0 or A260/230 1.8–2.2 via NanoDrop^TM^ 2000 spectrophotometer) were excluded from subsequent analyses. Additional exclusion criteria included inadequate RNA integrity. Outliers in spike-in Ct values were identified using Tukey's method: Ct values > 3 × IQR beyond Q1/Q3 quartiles (extreme outliers) were excluded as extraction/cDNA synthesis failures. Melting curve analysis showed non-specific amplification/primer dimers.

### 2.15 Technical replicates

All RT-qPCR reactions were performed in technical duplicates (FFPE tissue, platelets, plasma), with mean Ct values used for ΔΔCt calculations. To ensure technical reliability, duplicate Ct values were required to differ by no more than 1 cycle. Negative controls (no-template ddH_2_O) confirmed absence of contamination.

### 2.16 Statistical analysis

Data analysis was performed using Microsoft Excel and SPSS_v29.0 (IBM, Ehningen, Germany). Figures were created in SPSS_v29.0 and BioRender.com. RT-qPCR data were analyzed applying the 2^−ΔΔCt^ method [37]. RT-qPCR was performed in duplicates and mean Ct values were used for subsequent calculations. For the analysis of FFPE tissue samples, ΔCt values were first determined by normalizing the mean Ct values of the target miRNAs to those of the reference RNAs *RNU6-2* and *SNORD49A*. ΔΔCt values were calculated by comparing the ΔCt of tumor tissue to the corresponding ΔCt of adjacent non-tumor tissue from the same patient. In cases where adjacent non-tumor tissue was unavailable, the group-specific average ΔCt of available adjacent tissues (enchondroma, chondrosarcoma G1, G2, or G3) was used. Relative expression was calculated as fold change using the formula 2^−ΔΔCt^. Raw Ct-data are provided as (Suppl. Table 3). To compare relative expression levels between adjacent non-tumor and tumor tissues, the Wilcoxon signed-rank test was applied. Differences in relative expression among patient groups (enchondroma and chondrosarcoma G1/ACT, G2, G3) were assessed using the Kruskal-Wallis test, followed by the Mann-Whitney *U* test for pairwise comparisons. For plasma and platelet samples, data analysis followed a similar approach. ΔCt values were obtained by normalizing to the reference miRNAs miR-16-5p, miR-423-3p, and miR-let-7b-5p. The ΔΔCt values were calculated using the mean ΔCt of healthy participants as the reference. Kruskal-Wallis and Mann-Whitney *U* test were applied for statistical analysis of relative miR-expression in enchondroma and low-grade chondrosarcoma G1/ACT patients compared to healthy controls. Raw Ct values are supplied in Suppl. Tables 4 and 5. Outliers in spike-in Ct values from plasma samples were classified according to Tukey’s boxplot method, with mild outliers defined as values 1.5–3 times the interquartile range (IQR) beyond the quartiles. The evaluation of plasma miRNAs as biomarkers for prediction of enchondroma/chondrosarcoma G1/ACT was performed by receiver operating characteristic (ROC) curves and area under the curve (AUC) with 95% confidence interval. All analyses were exploratory and no correction for multiple testing was performed. P-value of *p < 0.05 was considered significant. Significance level of p < 0.01 was indicated as **p.

## 3 Results

### 3.1 Expression analysis of miR-138-5p, miR-181a-5p, miR-143-3p, and miR-145-5p in human FFPE tissues comparing tumor and adjacent non-tumor tissue from enchondroma and chondrosarcoma G1/ACT-G3

**Fig. 1:**
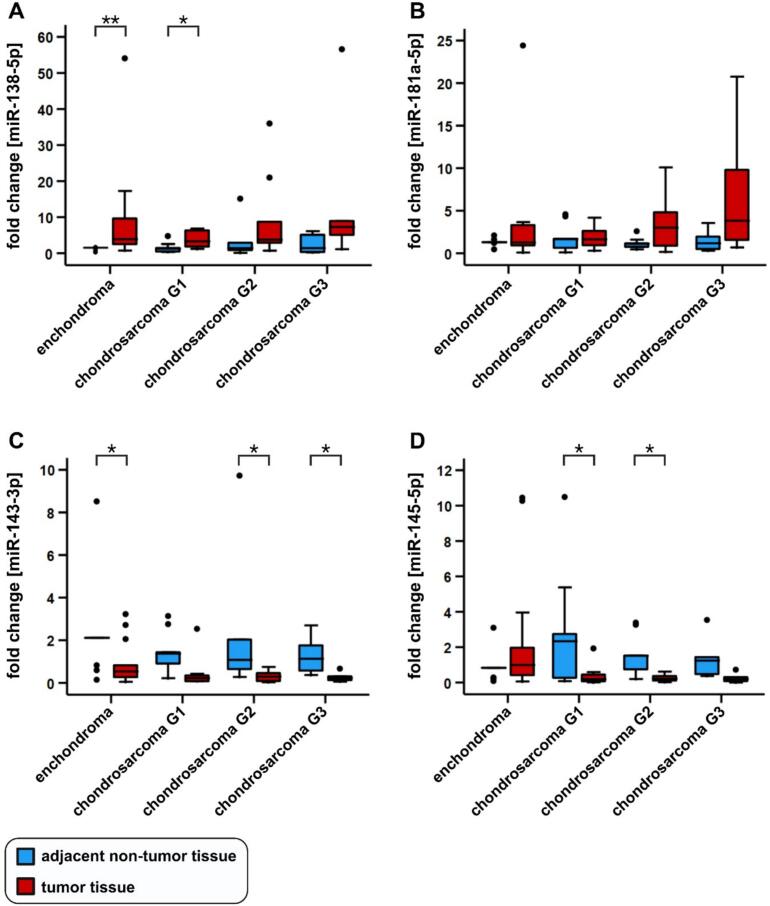
MiR-138-5p, miR-181a-5p, miR-143-3p, and miR-145-5p in human FFPE tissues. Quantitative expression analysis of miRNAs in human FFPE tumor- and adjacent non-tumor tissues from enchondroma (n = 16), low-grade chondrosarcoma G1/ACT (n = 11), chondrosarcoma G2 (n = 10), and chondrosarcoma G3 (n = 6) patients. MiR-138-5p- (A), miR-181a-5p- (B), miR-143-3p- (C), and miR-145-5p (D) relative expression in FFPE tissues of enchondroma and chondrosarcoma (G1-G3) patients compared to adjacent non-tumor tissues. Significance levels: *p < 0.05, **p < 0.01.

To identify miR-138-5p, miR-181a-5p, miR-143-3p, and miR-145-5p as biomarkers for chondrogenic tumors, expression analysis was performed in human FFPE tissues. Tissue from enchondroma (n = 16), low-grade chondrosarcoma G1/ACT (n = 11), and high-grade chondrosarcoma G2 (n = 10) and G3 (n = 6) patients was analyzed (Table 1). The expression of miR-138-5p was shown to be significantly increased in enchondroma tissue compared to the adjacent non-tumor tissue (fold change 2.56, IQR 7,39, times upregulation 0.39, **p = 0.002) and significantly in chondrosarcoma G1/ACT tissue compared to the adjacent non-tumor tissue (fold change 2.56, IQR 4.6, times upregulation 0.39, *p = 0.037), respectively (Table 3 and Fig. 1A). For miR-181a-5p no significant differences in expression were detected between all tumor tissues and the corresponding adjacent non-tumor tissue (Table 3 and Fig. 1B). A significant downregulation of miR-143-3p in tumor tissue was shown for the comparison of enchondroma (fold change 0.25, IQR 0.88, times downregulation 4, *p = 0.046), chondrosarcoma G2 (fold change 0.27, IQR 0.42, times downregulation 3.7, *p = 0.011), and chondrosarcoma G3 (fold change 0.19, times downregulation 5.2, IQR 0.29, *p = 0.046), respectively with adjacent non-tumor tissue. Chondrosarcoma G1/ACT and adjacent non-tumor tissue revealed no significantly discernible expression difference, however, a trend towards lower expression in chondrosarcoma G1/ACT was observed (p = 0.059, Table 3 and Fig. 1C). No significant differences in miR-145-5p expression were demonstrated between enchondroma and adjacent non-tumor tissue (p = 0.363). In contrast, chondrosarcoma G1/ACT and G2 tissues showed a significant decrease in miR-145-5p expression compared to adjacent non-tumor tissue (fold change 0.08, IQR 0.43, times downregulation 12.5, *p = 0.047 and fold change 0.14, IQR 0.28, times downregulation 7.14, *p = 0.012, Table 3 and Fig. 1D). These results demonstrate miR-138-5p, miR-143-3p, and miR-145-5p as potential biomarkers in tissue samples of chondrogenic tumors.

**Table 3:** Fold changes, interquartile ranges (IQR), and p-values of miR-138-5p, miR-181a-5p, miR-143-3p, and miR-145-5p in human FFPE tissue samples.

miRNA		miR- 138	miR- 181a	miR- 143	miR- 145
Enchondroma	fold change	2.56	0.97	0.25	1.19
	IQR	7.39	2.54	0.88	2.09
Chondrosarcoma G1/ACT	fold change	2.56	0.99	0.16	0.08
	IQR	4.60	1.94	0.29	0.43
Chondrosarcoma G2	fold change	2.64	2.66	0.27	0.14
	IQR	9.40	4.45	0.42	0.28
Chondrosarcoma G3	fold change	5.14	3.25	0.19	0.15
	IQR	16.75	11.18	0.29	0.35
p-value	adjacent vs. enchondroma	0.002**	0.397	0.046*	0.363
	adjacent vs. G1/ACT	0.037*	0.799	0.059	0.047*
	adjacent vs. G2	0.059	0.059	0.011*	0.012*
	adjacent vs. G3	0.075	0.116	0.046*	0.075

n = 43. Significance levels: *p < 0.05, **p < 0.01.

### 3.2 Comparison of miR-138-5p, miR-181a-5p, miR 143-3p, and miR-145-5p expression for their ability to differentiate enchondroma from chondrosarcoma G1/ACT-G3 in tissue

**Fig. 2:**
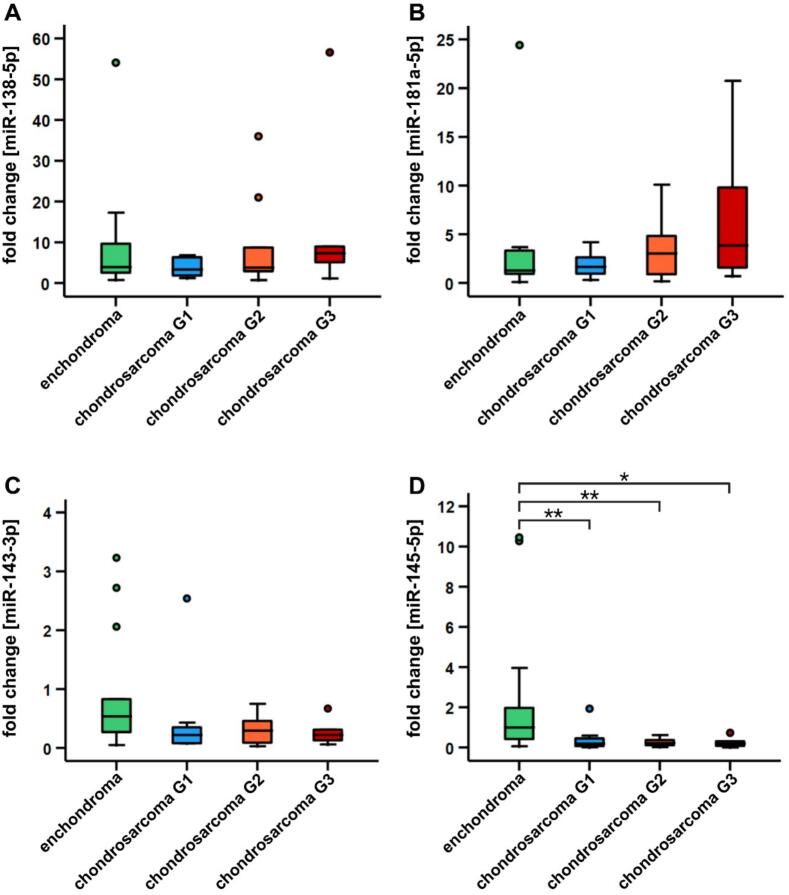
MiR-138-5p, miR-181a-5p, miR-143-3p, and miR-145-5p expression in enchondroma and chondrosarcoma grade 1/ACT. Comparative expression analysis of miR-138-5p, miR-181a-5p, miR-143-3p, and miR-145-5p in human FFPE tumor tissues between enchondroma and different grades of chondrosarcoma. Enchondroma (n = 16), low-grade chondrosarcoma G1/ACT (n = 11), chondrosarcoma G2 (n = 10), and chondrosarcoma G3 (n = 6) patients (A-D). No significant difference in expression was detected for miR-138-5p, miR-181a-5p, and miR-143-3p (A-C). In contrast, miR-145-5p showed significant differences in expression, with downregulation in chondrosarcoma G1/ACT-G3 compared to enchondroma tissues (D). No significant differences were found among the chondrosarcoma grades for miR-138-5p, miR-181a-5p, miR-143-3p, and miR-145-5p (A-D). Significance levels: *p < 0.05, **p < 0.01.

MiR-138-5p, miR-181a-5p, miR 143-3p, and miR-145-5p were evaluated for the capability to distinguish between enchondroma and different grades of chondrosarcoma in tissue (Table 4 and Fig. 2A–D). For miR-138-5p, no significant differences in expression were observed, comparing enchondroma with the different chondrosarcoma groups. Furthermore, no significant differences were detected between the three different grades of chondrosarcomas (Table 4 and Fig. 2A). Similar results were observed for miR-181a-5p, as expression levels did not differ significantly between enchondroma and the different chondrosarcoma grades as well as among the different chondrosarcoma grades, respectively (Table 4 and Fig. 2B). The comparison between enchondroma and chondrosarcoma grade 1/ACT for miR-143-3p showed a trend toward lower expression in chondrosarcoma G1/ACT (p = 0.053). No significant differences were detected among the different chondrosarcoma grades (Table 4 and Fig. 2C). However, for miR-145-5p, significant differences in expression were observed between enchondroma and all grades of chondrosarcoma. Compared to enchondroma, miR-145-5p expression was significantly downregulated in chondrosarcoma grade 1/ACT (fold change 0.19, IQR 0.43, times downregulation 5.26, **p = 0.008), chondrosarcoma grade 2 (fold change 0.22, IQR 0.28, times downregulation 4.55, **p = 0.007), and grade 3 chondrosarcoma (fold change 0.19, IQR 0.35, times downregulation 5.26, *p = 0.017). No significant differences were detected for miR-145-5p among the different chondrosarcoma grades (Table 4 and Fig. 2D).

**Table 4:** Fold changes (compared to enchondroma), interquartile ranges (IQR), and p-values for the expression analysis of miR-138-5p, miR-181a-5p, miR-143-3p, and miR-145-5p in human FFPE tissue samples.

miRNA		miR-138	miR-181a	miR-143	miR-145
Chondrosarcoma G1/ACT	fold change	0.85	1.30	0.41	0.19
	IQR	4.60	1.94	0.29	0.43
Chondrosarcoma G2	fold change	0.97	2.38	0.55	0.22
	IQR	9.40	4.45	0.42	0.28
Chondrosarcoma G3	fold change	1.87	3.03	0.41	0.19
	IQR	16.75	11.18	0.29	0.35
p-value	enchondroma vs. G1/ACT	0.266	1.000	0.053	0.008**
	enchondroma vs. G2	0.861	0.380	0.089	0.007**
	enchondroma vs. G3	0.458	0.138	0.083	0.017*
	chondrosarcoma G1/ACT vs. G2	0.762	0.820	0.820	0.677
	chondrosarcoma G1/ACT vs. G3	0.083	0.828	0.828	0.913
	chondrosarcoma G2 vs. G3	0.233	0.871	0.871	0.870

n = 43. Significance levels: *p < 0.05, **p < 0.01.

### 3.3 MiR-138-5p, miR-181a-5p, miR-143-3p, and miR-145-5p expression in human platelets

**Fig. 3:**
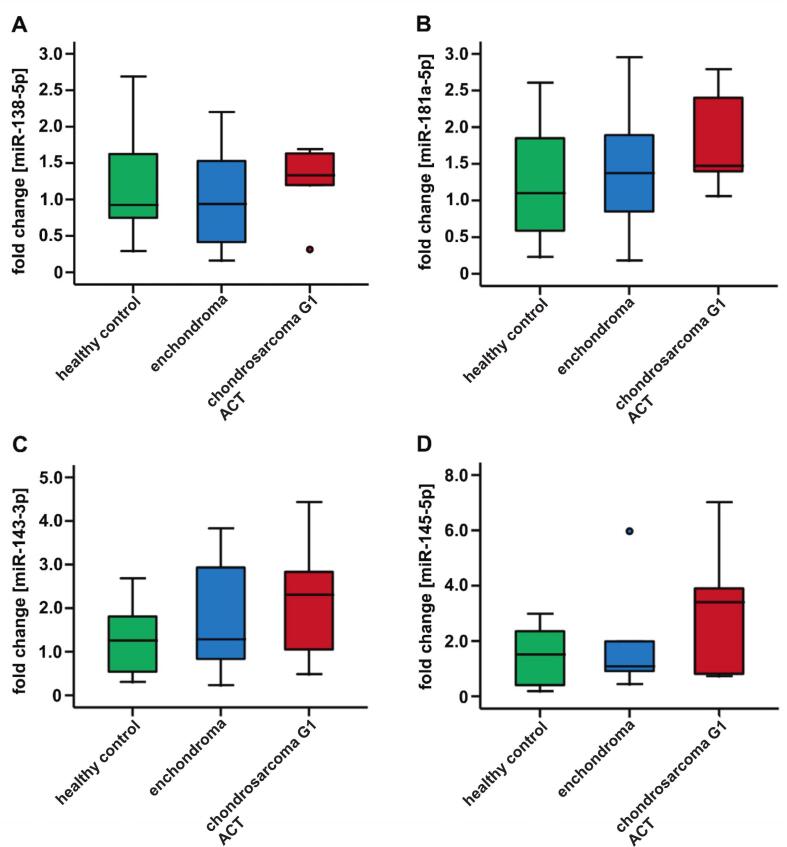
MiR-138-5p, miR-181a-5p, miR-143-3p, and miR-145-5p expression in human platelets. MiR-138-5p, miR-181a-5p, miR-143-3p, and miR-145-5p in human platelets from healthy participants (n = 12), enchondroma (n = 6), and low-grade chondrosarcoma G1/ACT (n = 6) patients. Relative expression of miR-138-5p (A), miR-181a-5p (B), miR-143-3p (C), and miR-145-5p (D) in human platelets. No significant differences between healthy controls and cartilaginous tumor patients or between enchondroma and low-grade chondrosarcoma G1/ACT patients was demonstrated. Significance levels: *p < 0.05, **p < 0.01.

To examine miRNAs derived from platelets for the discrimination between enchondroma and low-grade chondrosarcoma G1/ACT, the relative expression of miR-138-5p, miR-181a-5p, miR-143-3p, and miR-145-5p was analyzed in human platelets (Table 5 and Fig. 3A–D). Expression of miR-138-5p in platelets of healthy controls compared to platelets from enchondroma or low-grade chondrosarcoma G1/ACT patients as well as the comparison of platelets from enchondroma versus low-grade chondrosarcoma G1/ACT patients revealed no significant difference (p = 0.574, p = 1.000, and p = 0.749, Table 5 and Fig. 3A). Similarly, the expression levels of miR-181a-5p demonstrated no significant differences comparing platelets from healthy controls to platelets from enchondroma and low-grade chondrosarcoma G1/ACT patients or platelets from enchondroma versus low-grade chondrosarcoma G1/ACT patients (p = 0.708, p = 0.349, and p = 0.749, Table 5 and Fig. 3B). For miR-143-3p and miR-145-5p, similar expression patterns were observed (Table 5 and Fig. 3C and D). Overall, the expression patterns of all four miRNAs in platelets did not demonstrate significant differences between healthy controls, enchondroma, and low- grade chondrosarcoma G1/ACT patients. Furthermore, no evidence for significant differences of miR-138-5p, miR-181a-5p, miR-143-3p, and miR-145-5p expression from platelets of enchondroma and low-grade chondrosarcoma G1/ACT were found.

**Table 5:** Fold changes, interquartile ranges (IQR), and p-values for the expression of miR-138-5p, miR-181a-5p, miR-143-3p, and miR-145-5p in human platelets.

miRNA		miR-138	miR-181a	miR-143	miR-145
Enchondroma	fold change	0.94	1.37	1.28	1.09
	IQR	1.34	1.47	2.47	2.19
Chondrosarcoma G1/ACT	fold change	1.33	1.47	2.31	3.40
	IQR	0.91	1.37	2.87	4.69
p-values	healthy vs. enchondroma	0.574	0.708	0.640	0.851
	healthy vs. chondrosarcoma G1/ACT	0.527	0.206	0.171	0.092
	enchondroma vs. chondrosarcoma G1/ACT	0.465	0.465	0.715	0.584

Healthy controls (n = 12), enchondroma patients (n = 6) and low-grade chondrosarcoma G1/ACT patients (n = 5), total n = 23. Significance levels: *p < 0.05, **p < 0.01.

### 3.4 MiR-138-5p, miR-181a-5p, miR-143-3p, and miR-145-5p expression in human plasma

**Fig. 4:**
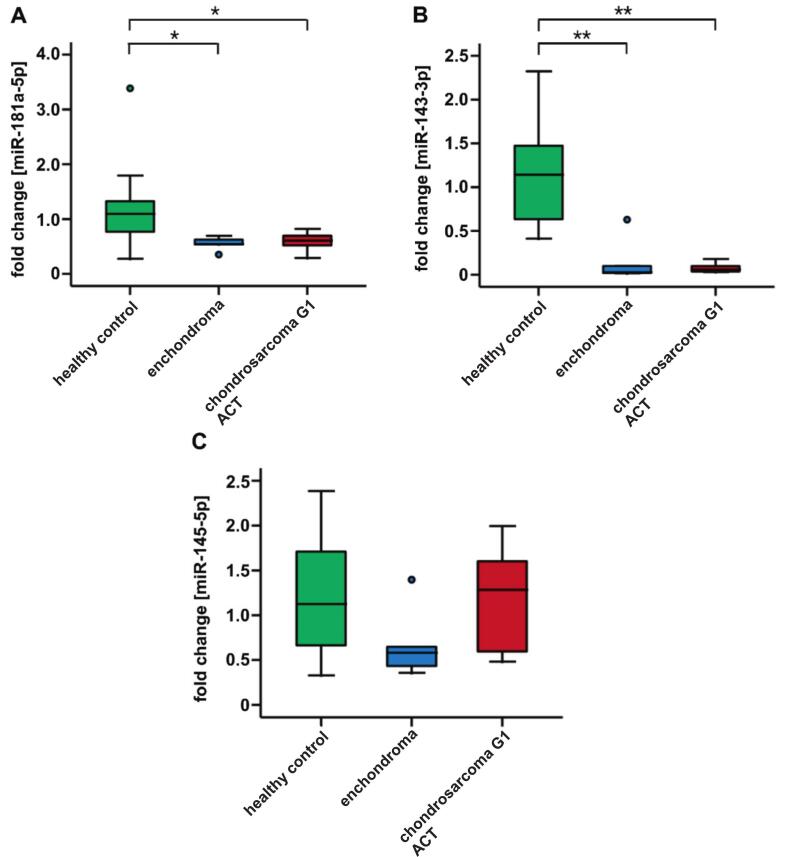
MiR-181a-5p, miR-143-3p, and miR-145-5p expression in human plasma. Evaluation of miR-181a-5p, miR-143-3p, and miR-145-5p in human plasma from healthy participants (n = 12), enchondroma (n = 6), and low-grade chondrosarcoma G1/ACT (n = 5) patients. MiR-181a-5p (A), miR-143-3p (B), and miR-145-5p (C) in human plasma. MiR-138-5p showed no expression in human plasma. Significant downregulation of miR-181a-5p and miR-143-3p was revealed in enchondroma and low-grade chondrosarcoma G1/ACT patients compared to healthy controls. No significant differences were observed between the tumor groups. MiR-145-5p showed no significant variation among the groups. Significance levels: *p < 0.05, **p < 0.01.

To investigate the potential of circulating miRNAs as biomarkers to distinguish enchondroma from low-grade chondrosarcoma G1/ACT in liquid biopsies, the expression levels of miR-138-5p, miR-181a-5p, miR-143-3p, and miR-145-5p were analyzed in human plasma. MiR-138-5p expression was not detected in any of the plasma samples tested. In contrast, miR-181a-5p, miR-143-3p, and miR-145-5p were identified in plasma. MiR-181a-5p was significantly downregulated in enchondroma and low-grade chondrosarcoma G1/ACT patients compared to healthy controls. The direct comparison between healthy controls and enchondroma patients showed a significant 1.9-times downregulation (fold change 0.54, times downregulation 1.9, IQR 0.15, *p = 0.019) of miR-181a-5p in enchondroma patients. In low-grade chondrosarcoma G1/ACT patients the expression was reduced by about 2.1-times (fold change 0.61, times downregulation 2.1, IQR 0.35, *p = 0.045). No significant differences were observed between enchondroma and low-grade chondrosarcoma G1/ACT patients, p = 0.715 (Table 6 and Fig. 4A). MiR-143-3p showed significant differential downregulation in expression between healthy controls and patients with enchondroma (fold change 0.02, times downregulation 33.3, IQR 0.21, **p = 0.002) or low-grade chondrosarcoma G1/ACT (fold change 0.06, times downregulation 16.7, IQR 0.11, **p = 0.002), respectively, with no significant difference in expression between enchondroma and low-grade chondrosarcoma G1/ACT with p = 0.234 (Table 6 and Fig. 4B). MiR-145-5p demonstrated no significant differences in relative expression in enchondroma or low-grade chondrosarcoma G1/ACT compared to healthy controls (p = 0.111 and p = 0.916). Moreover, miR-145-5p levels showed no significant differences in enchondroma compared to low-grade chondrosarcoma G1/ACT patients (p = 0.201, Table 6 and Fig. 4C). Altogether, plasma analysis revealed significant downregulation of miR-181a-5p and miR-143-3p in enchondroma and low-grade chondrosarcoma G1/ACT patients compared to healthy controls. Although discrimination between healthy controls and enchondroma as well as low-grade chondrosarcoma G1/ACT was possible, no significant differences were observed between the tumor groups.

**Table 6:** Fold changes, interquartile ranges (IQR), and p-values for expression analysis of miR-181a-5p, miR-143-3p, and miR-145-5p in human plasma.

miRNA		miR-181a	miR-143	miR-145
Enchondroma	fold change	0.54	0.03	0.58
	IQR	0.15	0.21	0.42
chondrosarcoma G1/ACT	fold change	0.61	0.06	1.28
	IQR	0.35	0.11	1.26
p-values	healthy vs. enchondroma	0.019*	0.002**	0.111
	healthy vs. chondrosarcoma G1/ACT	0.045*	0.002**	0.916
	enchondroma vs. chondrosarcoma G1/ACT	0.715	0.234	0.201

Healthy controls (n = 12), enchondroma patients (n = 6) and low-grade chondrosarcoma G1/ACT patients (n = 5), total n = 23. Significance levels: *p < 0.05, **p < 0.01.

### 3.5 Diagnostic accuracy of plasma miR-181a-5p and miR-143-3p in differentiating enchondroma from low-grade chondrosarcoma G1/ACT

**Fig. 5:**
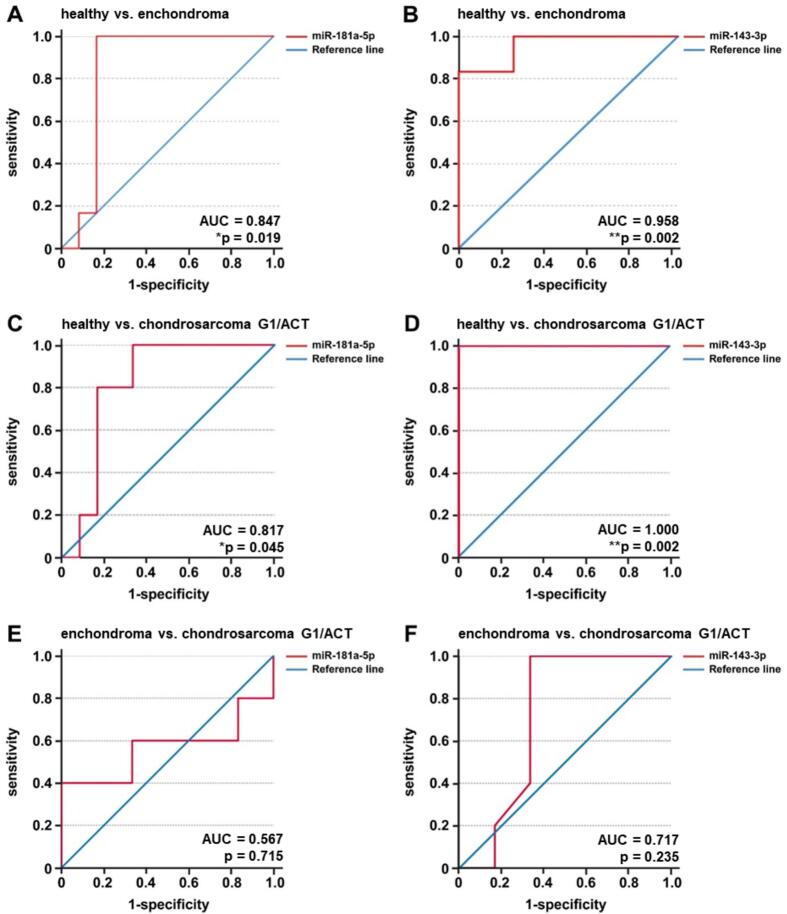
ROC curve analysis of miR-181a-5p and miR-143-3p in plasma. Receiver operating characteristic (ROC) curves for miR-181a-5p and miR-143-3p in plasma samples of enchondroma patients, low-grade chondrosarcoma G1/ACT patients and healthy controls. MiR-181a-5p and miR-143-3p significantly discriminated healthy controls from enchondroma patients (A and B). Similarly, both miRNAs showed significant discriminatory power between healthy controls and low-grade chondrosarcoma G1/ACT patients (C and D). In contrast, ROC analysis between enchondroma and low-grade chondrosarcoma G1/ACT patients revealed no significant discrimination for miR-181a-5p and miR-143-3p, respectively (E and F). Significance levels: *p < 0.05, **p < 0.01.

Following the differential expression analysis, receiver operating characteristic (ROC) curve analysis was conducted to assess the ability of miR-181a-5p and miR-143-3p to discriminate between healthy controls, enchondroma, and low-grade chondrosarcoma G1/ACT patients. Comparing healthy individuals to enchondroma patients, miR-181a-5p demonstrated an area under the curve (AUC) of 0.847, 95% confidence interval 0.65–1.000, *p = 0.019 (Fig. 5A), while miR-143-3p reached an AUC of 0.958, 95% confidence interval 0.866–1.000, **p = 0.002, indicating significant accuracy (Fig. 5B). Similarly, both miRNAs showed discriminatory power with significance in distinguishing healthy controls from low-grade chondrosarcoma G1/ACT patients, with AUC values of 0.817 for miR-181a-5p, 95% confidence interval 0.611–1.000, *p = 0.045 (Fig. 5C) and AUC 1.000 for miR-143-3p, 95% confidence interval 1.000–1.000, **p = 0.002 (Fig. 5D), respectively. In contrast, ROC analysis comparing enchondroma and low-grade chondrosarcoma G1/ACT patients revealed no significant discriminatory ability. For miR-181a-5p, the AUC was 0.567 with 95% confidence interval 0.177–0.957, p = 0.715. MiR-143-3p had an AUC of 0.717 with 95% confidence interval 0.377–1.000, p = 0.235 (Fig. 5E and F).

### 3.6 Proposed miRNA-guided workflow for clinical practice

**Fig. 6:**
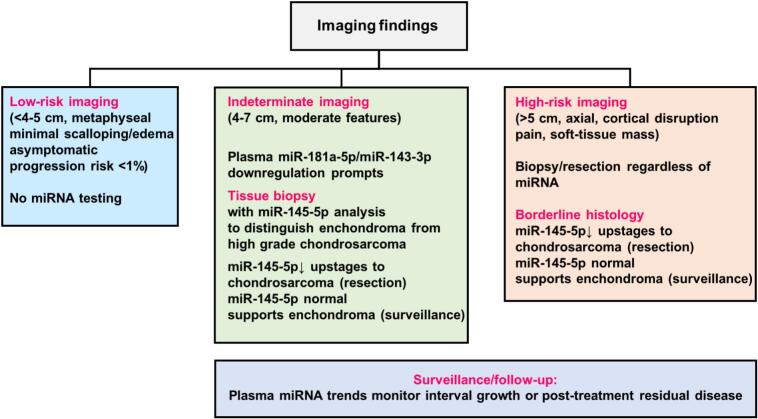
MiRNA-guided clinical workflow. Schematic representation of the proposed miRNA–guided diagnostic workflow for distinguishing enchondroma from chondrosarcoma. The algorithm integrates plasma miRNA profiling (miR–181a–5p, miR–143–3p) and tissue–specific miRNA evaluation (miR–145–5p) as complementary tools into imaging–based risk stratification (RAS/BACTIP).

To enhance translational applicability, we explored the potential integration of miR-145–5p expression in tissue, as well as miR-181a-5p and miR-143-3p expression in plasma, into existing diagnostic workflows. These minimally invasive miRNA assays may serve to complement conventional biopsy, particularly in cases presenting with imaging‑indeterminate lesions (e.g., long‑bone lesions measuring 4–7  cm with moderate endosteal scalloping and edema) or with borderline histopathological findings where enchondroma and low‑grade chondrosarcoma (G1/ACT) exhibit overlapping morphological features. In this model, lesions with low-risk imaging characteristics (e.g., <4–5  cm, metaphyseal location, minimal scalloping, absence of edema, and asymptomatic presentation according to RAS/BACTIP) do not require miRNA testing, given the negligible progression risk (<1%). In contrast, high-risk imaging features (e.g., >5 cm, axial location, cortical disruption, pain, or soft-tissue involvement) warrant biopsy or surgical resection irrespective of miRNA results. For cases with borderline histopathology, downregulation of miR-145-5p supports upstaging to chondrosarcoma and indication for resection, whereas normal miR-145–5p expression favors an enchondroma diagnosis and surveillance strategy. For indeterminate imaging findings (4–7  cm lesions with moderate radiologic features), plasma-based assessment of miR-181a-5p and miR-143-3p indicates chondrogenic tumor. Downregulation of these markers prompts tissue biopsy with testing for miR-145-5p. During follow-up, serial plasma miRNA measurements allow monitoring of interval growth or residual disease after resection (Fig. 6). Further studies in independent cohorts are required for validation of this model and integration into clinical practice.

## 4 Discussion

Despite advances in imaging and pathologic diagnostics, differentiation in chondrogenic tumors between benign enchondroma and malignant chondrosarcoma remains difficult due to overlapping features [38]. This distinction is clinically important, as it directly affects surveillance and treatment decisions with potential influence on patient outcomes [17]. Even with histopathological analysis, diagnostic inconsistency persists. This was demonstrated by Eefting et al. who reported only moderate interobserver agreement (κ = 0.54) among expert pathologists for the differentiation of enchondroma from chondrosarcoma [16]. Particularly challenging remains the distinction between enchondroma and low-grade chondrosarcoma G1/ACT. In clinical practice this difficulty may lead to disproportional procedures and high frequency follow-up monitoring, including invasive tumor biopsies and repetitive imaging to ensure adequate surveillance. Considering the diagnostic limitations, interest in molecular biomarkers such as miRNAs is increasing, as they are easily accessible and offer potential for more accurate diagnostics [34]. Although various biomarkers have been introduced in the past decades, no reliable and standardized biomarkers are available for clinical use [23].

This work aimed to investigate the potential of miR-138-5p, miR-181a-5p, miR-143-3p, and miR-145-5p as biomarkers for the diagnosis of enchondroma and chondrosarcoma in tissue biopsies and the differentiation of enchondroma from low-grade chondrosarcoma G1/ACT in tissue and liquid biopsies. These candidate miRNAs were selected based on prior studies, which suggested their relevance in the discrimination of chondrogenic tumors [35], [36]. Zhang et al. demonstrated a significant upregulation of miR-138-5p and miR-181a-5p in human FFPE low-grade chondrosarcoma compared to enchondroma samples [35]. Urdinez et al. found significant downregulation of miR-143-3p and miR-145-5p in human FFPE chondrosarcoma compared to enchondroma tissues. Particularly, miR-145-5p showed promise in distinguishing low-grade chondrosarcoma from enchondroma. Additionally, the same study analyzed the expression patterns of both miRNAs in human plasma. Statistically significant differences in miR-145-5p expression were identified between enchondroma and high-grade chondrosarcoma (grades 2 and 3) [36].

Comparison of miRNA expression in our study in tissues from chondrogenic tumors with adjacent non-tumor tissues revealed a significant upregulation of miR-138-5p in enchondroma and low-grade chondrosarcoma G1/ACT tissues compared to adjacent non-tumor tissues. For miR-181a-5p, a trend toward higher expression in high-grade chondrosarcoma was observed, but the differences were not statistically significant. Zhang et al. reported higher expression levels of both miR-138-5p and miR-181a-5p in low-grade chondrosarcoma. Compared to enchondromas, their expression was approximately 7-fold higher, and nearly 100-fold higher compared to normal cartilage. Based on these findings, the authors proposed both miRNAs as promising biomarkers for distinguishing enchondroma from low-grade chondrosarcoma [35]. However, our study, which included a larger and more differentiated FFPE tissue cohort, did not replicate these exact findings. In our study we did not find higher expression of miR-138-5p or miR-181a-5p in low-grade chondrosarcoma G1/ACT compared to enchondroma. Several factors may account for the discrepancies between the two studies. Firstly, our study analyzed a larger cohort of 43 FFPE tissue samples, including a separation of chondrosarcomas by histological grade (G1/ACT, G2, and G3). In contrast, Zhang et al. included fewer samples with 6 enchondroma and 8 chondrosarcoma patients [35]. The grade-specific analysis in our study allowed for a more detailed view of expression patterns across tumor progression. Another important methodological difference is the choice of control tissue. While Zhang et al. used healthy cartilage, our study used adjacent non-tumor tissue from the same patient, minimizing interpatient and site-specific variability [39].

The observed upregulation of miR-138-5p in enchondroma and low-grade chondrosarcoma G1/ACT compared to adjacent non-tumor tissue aligns with its known biological function reported in earlier research [40]. Seidl et al. suggest a role of miR-138-5p in chondrocyte dedifferentiation by targeting SP1 and HIF-2α, which are key transcription factors for cartilage homeostasis. Both transcription factors promote the expression of COL2A1, the major type II collagen in articular cartilage, which is important for normal cartilage function [46]. The observed increase of miR-138-5p in enchondroma and low-grade chondrosarcoma G1/ACT may therefore contribute to early chondrocyte dedifferentiation during cancer progression by the suppression of SP1 and HIF-2α [40]. Although this study did not identify miR-181a-5p as a biomarker for differentiation between chondrogenic tumors and adjacent non-tumor tissue, it may have biological function in tumor development. In chondrosarcoma, it has been characterized predominantly as an oncomiRNA. A study by Sun et al. demonstrated that miR-181a-5p is hypoxia regulated and enhances VEGF expression, suggesting a role in promoting angiogenesis and tumor progression in chondrosarcoma [41].

Conversely, miR-143-3p and miR-145-5p were downregulated in chondrogenic tumor tissues. MiR-143-3p was significantly downregulated in enchondroma as well as in chondrosarcoma G2 and G3 tissues compared to adjacent non-tumor tissues. The difference in low-grade chondrosarcoma G1/ACT was borderline significant (p = 0.059) and may reach significance with higher sample size. Similarly, miR-145-5p was significantly downregulated in low-grade chondrosarcoma G1/ACT and G2 tissues compared to adjacent non-tumor tissue. While its reduction in chondrosarcoma G3 did not meet the significance threshold (p = 0.075), the consistent trend across all chondrosarcoma grades supports its potential utility as a diagnostic marker. MiR-145-5p was significantly downregulated in all chondrosarcoma grades compared to enchondroma, reinforcing its potential as a reliable marker to differentiate enchondroma from chondrosarcoma in tissue.

Both miR-143-3p and miR-145-5p have well-established roles as tumor suppressors, exerting biological effects that are critical in chondrogenic tumor progression [36], [42]. Their downregulation leads to the derepression of oncogenic targets such as FSCN1, a protein involved in cell motility and invasion [[32], [43], [44]]. In chondrosarcoma, elevated FSCN1 levels are associated with advanced tumor grade and its depletion reduced the migratory capacity of cells [36]. Therefore, the observed reduced expression patterns of these miRNAs not only serve as diagnostic biomarkers but also reflect underlying molecular mechanisms contributing to tumor progression [36]. The findings of this study underscore the dual importance of miR-143-3p and miR-145-5p as both biomarkers and functional regulators in chondrogenic tumors.

Tumor-educated platelets (TEPs) have been demonstrated to carry cancer-specific miRNA profiles, suggesting their utility as biomarkers for cancer diagnosis [45]. In this analysis we investigated the diagnostic potential of miR-138-5p, miR-181a-5p, miR-143-3p, and miR-145-5p in human platelets for detecting chondrogenic tumors and discrimination of enchondroma versus low-grade chondrosarcoma G1/ACT. Whereas miR-138-5p was expressed in tissue samples it was not detected in platelets. None of the tested miRNAs showed significant differences in expression levels between healthy controls, enchondroma, and low-grade chondrosarcoma G1/ACT patients. These findings contrast with our plasma and FFPE tissue data, which revealed significant miRNA expression changes. This discrepancy likely reflects fundamental biological and physiological characteristics of enchondromas and low-grade chondrosarcomas G1/ACT. Both tumor types are characterized by low vascularity and a predominantly localized growth pattern, resulting in limited systemic release of tumor-derived factors. Consequently, platelet-tumor interactions and the process of platelet “education” may be minimal in these entities. Furthermore, platelet education is known to be tumor-specific [46] and the relatively small tumor burden in our patient cohort may contribute to the low abundance of circulating tumor signals capable of altering platelet miRNA composition. In contrast, soluble miRNAs in plasma may more readily capture subtle tumor-associated molecular changes, even in biologically indolent lesions. These findings suggest that the systemic effects of enchondromas and low-grade chondrosarcomas are modest and may not suffice to imprint specific miRNA signatures onto circulating platelets. Moreover, the detection of miR-181a-5p, miR-143-3p, and miR-145-5p in plasma but not in platelets may reflect differences in the biological origin, release mechanisms, and turnover of circulating RNA species. Plasma miRNAs are derived from multiple sources, including tumor cells, stromal cells, and the surrounding microenvironment, and are often secreted in extracellular vesicles or bound to proteins such as Argonaute-2, which protect them from degradation [47]. In contrast, platelet miRNA profiles are shaped through active interactions with tumor-derived factors and depend on the extent of platelet “education” [48]. For enchondromas and low-grade chondrosarcomas G1/ACT, both the low vascularization and limited systemic signaling likely impede the uptake of tumor-derived miRNAs by circulating platelets. The localized bone and cartilage microenvironment may further restrict direct platelet-tumor contact. Consequently, tumor-associated miRNAs may be released into the circulation in soluble or vesicular form, accounting for their detectability in plasma, while platelet-associated transfer remains negligible. Brinkman et al. 2020 analyzed blood from twelve melanoma patients with confirmed BRAF V600E mutations. The extracellular vesicle fraction showed mutant BRAF in ten patients, while platelet fractions contained only wild-type BRAF without detectable mutation signals [49], [50]. Thus, plasma may be a more sensitive compartment for detecting subtle molecular changes in indolent or poorly vascularized chondrogenic tumors. Future studies with larger and more heterogeneous cohorts, including high-grade and more vascularized chondrosarcomas, are warranted to further elucidate tumor-platelet communication in chondrogenic neoplasms.

To assess their diagnostic value in liquid biopsies, the expression levels of miR-138-5p, miR-181a-5p, miR-143-3p, and miR-145-5p were measured in plasma samples. MiR-138-5p was not detectable in human plasma, suggesting low or absent circulating levels rather than technical failure. This was supported by the detection of miR-138-5p in enchondroma and low-grade chondrosarcoma G1/ACT tissue samples, confirming assay validity. The low abundance of many miRNAs in biofluids represents a current challenge in biomarker research, complicating their reliable detection. In contrast, a significant downregulation of miR-181a-5p and miR-143-3p in plasma from patients with enchondroma and low-grade chondrosarcoma G1/ACT compared to healthy controls was revealed. However, no statistically significant differences were observed between enchondroma and low-grade chondrosarcoma G1/ACT patients. These findings suggest that both miR-181a-5p and miR-143-3p may serve as general diagnostic biomarkers for the presence of chondrogenic tumors. In addition to their differential expression, miR-181a-5p and miR-143-3p demonstrated significant diagnostic potential based on ROC curve analysis, respectively. Both miRNAs were able to significantly distinguish patients with enchondroma or low-grade chondrosarcoma G1/ACT from healthy controls, with miR-143-3p showing particularly high accuracy. These values indicate a high diagnostic performance, underscoring their potential as minimal invasive biomarkers. The small sample size limits definitive conclusions, however, the results underline the potential of miR-181a-5p and miR-143-3p as promising biomarkers in liquid biopsies. A larger cohort may reveal statistically significant differences and better define the utility of miR-143-3p in the discrimination between enchondroma and low-grade chondrosarcoma G1/ACT.

To improve translational applicability, the potential integration of miR–138–5p, miR–143–3p, and miR–145–5p in tissue, together with miR–181a–5p and miR–143–3p in plasma, was evaluated within established diagnostic frameworks. This conceptual algorithm positions miRNA profiling as a rapid, non-invasive adjunct to imaging and conventional biopsy, particularly in imaging–indeterminate lesions (e.g., long–bone lesions measuring 4–7  cm with moderate endosteal scalloping and edema) or in borderline histopathological contexts where enchondroma and low–grade chondrosarcoma (G1/ACT) share overlapping morphological characteristics. Its use may reduce overtreatment of indolent enchondromas and expedite detection of malignant transformation. The approach is particularly relevant for the intermediate–risk group, in whom conservative management is frequently limited by functional or structural compromise requiring intervention. Prospective, multicenter validation integrating RAS, BACTIP, and radiomics frameworks will be necessary to define robust sensitivity and specificity thresholds for routine clinical implementation.

In conclusion, our findings suggest that miR–138–5p, miR–181a–5p, miR–143–3p, and miR–145–5p may represent promising diagnostic biomarkers in patients with chondrogenic tumors and indicate that combination of several miRNAs could increase diagnostic performance. To substantiate these observations and better define their clinical utility, future studies should validate our results in larger, independent cohorts. Additionally, further work should examine their incorporation into multiparametric diagnostic panels alongside additional molecular and imaging markers to refine diagnostic accuracy.

## CRediT authorship contribution statement

**Annabell Walter:** Writing – original draft, Visualization, Validation, Methodology, Investigation, Formal analysis, Data curation. **Kathrin Katenkamp:** Methodology. **Nikolaus Gaßler:** Methodology. **Thomas Lehmann:** Formal analysis. **Wolfram Weschenfelder:** Methodology. **Christian Spiegel:** Methodology. **Gunther O. Hofmann:** Writing – review & editing. **Amer Malouhi:** Writing – review & editing. **Andreas Hochhaus:** Writing – review & editing. **Jenny Rinke:** Writing – review & editing. **Joachim H. Clement:** Writing – review & editing, Methodology. **Karin G. Schrenk:** Writing – review & editing, Writing – original draft, Supervision, Conceptualization.

## Ethics approval and consent to participate

The study was approved by the institutional review board of the Ethics Committee of the Friedrich-Schiller-Universität Jena with Reg. Nr. 2024-3339_1-Material on July 17, 2024 and was conducted in accordance with the Declaration of Helsinki. Informed consent for the use of tissue sections was obtained from 24 patients or their relatives. 19 patients could not be reached because they had moved or died and no family contact was available. Informed consent was obtained from all healthy participants and patients donating blood samples.

## Funding

The authors declare that no financial support was received for the research and/or publication of this article.

## Declaration of competing interest

The authors declare that they have no known competing financial interests or personal relationships that could have appeared to influence the work reported in this paper.
