# Supplementary material for: MiRNA-based expression signatures in differential diagnosis of enchondroma and chondrosarcoma
Source: J Bone Oncol. 2026 Apr 8;58:100761. doi: 10.1016/j.jbo.2026.100761 (PMC13141744; doi:10.1016/j.jbo.2026.100761)
Supplement: Supplementary Data 1 [file mmc1.docx]

**Suppl. Table 1**

Primers used for detection of microRNAs in FFPE tissues and
thrombocytes of enchondroma and chondrosarcoma patients.

| **Primer ID** | **Catalog number** |
| --- | --- |
| hsa-miR-138-5p | HmiRQP0177 |
| hsa-miR-181a-5p | HmiRQP0232 |
| hsa-miR-143-3p | HmiRQP0188 |
| hsa-miR-145-5p | HmiRQP0192 |
| hsa-let-7b-5p | HmiRQP0004 |
| hsa-miR-16-5p | HmiRQP0227 |
| hsa-miR-423-3p | HmiRQP0491 |
| RNU6-2 | HmiRQP9001 |
| SNORD49A | HmiRQP9061 |

**Suppl. Table 2**

Primers used for expression analysis of microRNAs in plasma
of enchondroma and chondrosarcoma patients.

| **Primer ID** | **Catalog number** |
| --- | --- |
| hsa-miR-138-5p | YP00206078 |
| hsa-miR-181a-5p | YP00206081 |
| hsa-miR-143-3p | YP00205992 |
| hsa-miR-145-5p | YP00204483 |
| hsa-miR-16-5p | YP00205702 |
| hsa-miR-30e-5p | YP00204714 |
| hsa-miR-93-5p | YP00204715 |
| UniSp2 | YP00203950 |
| UniSp6 | YP00203954 |
