# Supplementary material for: MiRNA-based expression signatures in differential diagnosis of enchondroma and chondrosarcoma
Source: J Bone Oncol. 2026 Apr 8;58:100761. doi: 10.1016/j.jbo.2026.100761 (PMC13141744; doi:10.1016/j.jbo.2026.100761)
Supplement: Supplementary Data 2 [file mmc2.pptx]

## Slide 1
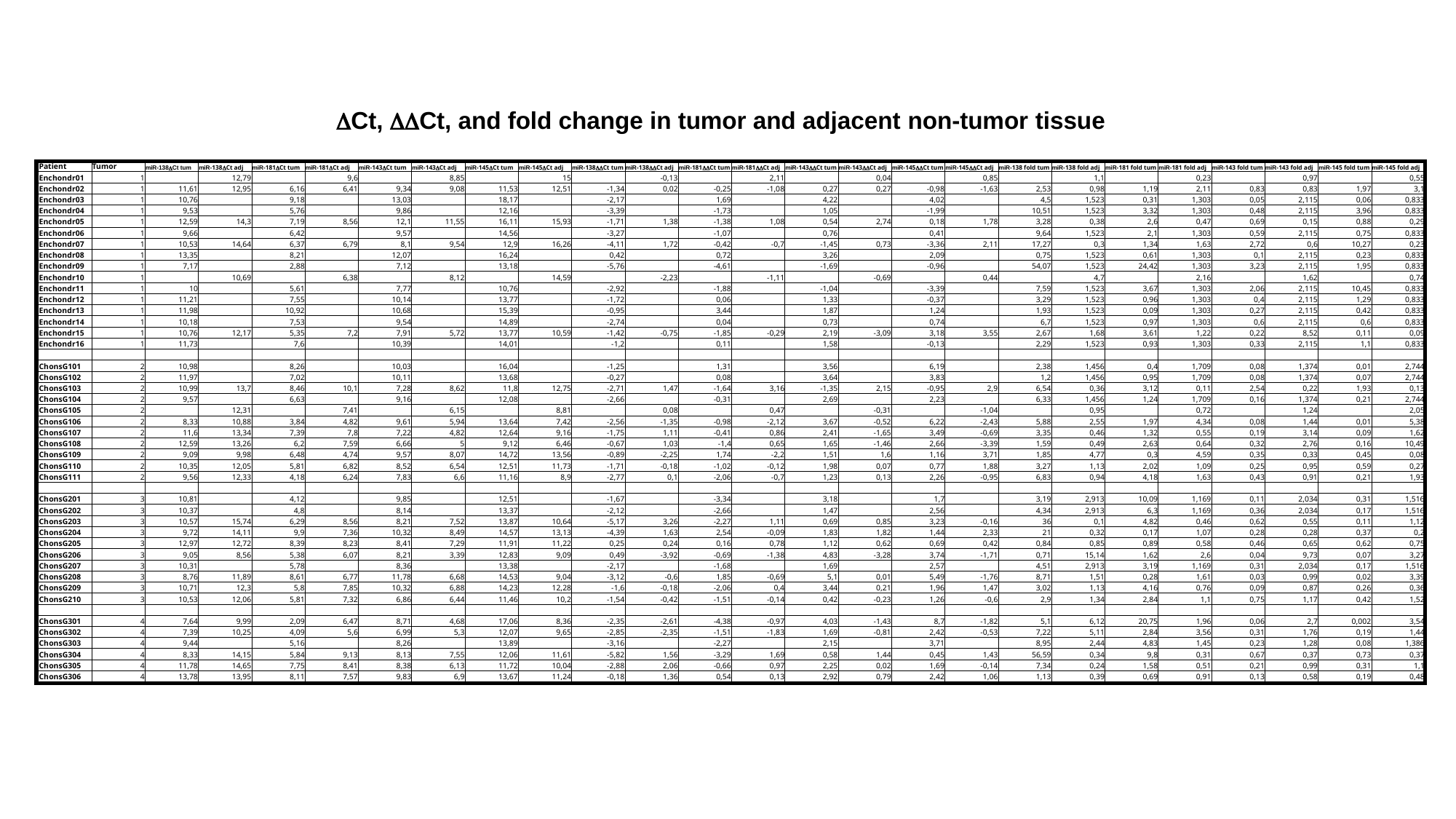

DCt, DDCt, and fold change in tumor and adjacent non-tumor tissue
| Patient | Tumor | miR-138DCt tum | miR-138DCt adj | miR-181DCt tum | miR-181DCt adj | miR-143DCt tum | miR-143DCt adj | miR-145DCt tum | miR-145DCt adj | miR-138DDCt tum | miR-138DDCt adj | miR-181DDCt tum | miR-181DDCt adj | miR-143DDCt tum | miR-143DDCt adj | miR-145DDCt tum | miR-145DDCt adj | miR-138 fold tum | miR-138 fold adj | miR-181 fold tum | miR-181 fold adj | miR-143 fold tum | miR-143 fold adj | miR-145 fold tum | miR-145 fold adj |
| --- | --- | --- | --- | --- | --- | --- | --- | --- | --- | --- | --- | --- | --- | --- | --- | --- | --- | --- | --- | --- | --- | --- | --- | --- | --- |
| Enchondr01 | 1 | | 12,79 | | 9,6 | | 8,85 | | 15 | | -0,13 | | 2,11 | | 0,04 | | 0,85 | | 1,1 | | 0,23 | | 0,97 | | 0,55 |
| Enchondr02 | 1 | 11,61 | 12,95 | 6,16 | 6,41 | 9,34 | 9,08 | 11,53 | 12,51 | -1,34 | 0,02 | -0,25 | -1,08 | 0,27 | 0,27 | -0,98 | -1,63 | 2,53 | 0,98 | 1,19 | 2,11 | 0,83 | 0,83 | 1,97 | 3,1 |
| Enchondr03 | 1 | 10,76 | | 9,18 | | 13,03 | | 18,17 | | -2,17 | | 1,69 | | 4,22 | | 4,02 | | 4,5 | 1,523 | 0,31 | 1,303 | 0,05 | 2,115 | 0,06 | 0,833 |
| Enchondr04 | 1 | 9,53 | | 5,76 | | 9,86 | | 12,16 | | -3,39 | | -1,73 | | 1,05 | | -1,99 | | 10,51 | 1,523 | 3,32 | 1,303 | 0,48 | 2,115 | 3,96 | 0,833 |
| Enchondr05 | 1 | 12,59 | 14,3 | 7,19 | 8,56 | 12,1 | 11,55 | 16,11 | 15,93 | -1,71 | 1,38 | -1,38 | 1,08 | 0,54 | 2,74 | 0,18 | 1,78 | 3,28 | 0,38 | 2,6 | 0,47 | 0,69 | 0,15 | 0,88 | 0,29 |
| Enchondr06 | 1 | 9,66 | | 6,42 | | 9,57 | | 14,56 | | -3,27 | | -1,07 | | 0,76 | | 0,41 | | 9,64 | 1,523 | 2,1 | 1,303 | 0,59 | 2,115 | 0,75 | 0,833 |
| Enchondr07 | 1 | 10,53 | 14,64 | 6,37 | 6,79 | 8,1 | 9,54 | 12,9 | 16,26 | -4,11 | 1,72 | -0,42 | -0,7 | -1,45 | 0,73 | -3,36 | 2,11 | 17,27 | 0,3 | 1,34 | 1,63 | 2,72 | 0,6 | 10,27 | 0,23 |
| Enchondr08 | 1 | 13,35 | | 8,21 | | 12,07 | | 16,24 | | 0,42 | | 0,72 | | 3,26 | | 2,09 | | 0,75 | 1,523 | 0,61 | 1,303 | 0,1 | 2,115 | 0,23 | 0,833 |
| Enchondr09 | 1 | 7,17 | | 2,88 | | 7,12 | | 13,18 | | -5,76 | | -4,61 | | -1,69 | | -0,96 | | 54,07 | 1,523 | 24,42 | 1,303 | 3,23 | 2,115 | 1,95 | 0,833 |
| Enchondr10 | 1 | | 10,69 | | 6,38 | | 8,12 | | 14,59 | | -2,23 | | -1,11 | | -0,69 | | 0,44 | | 4,7 | | 2,16 | | 1,62 | | 0,74 |
| Enchondr11 | 1 | 10 | | 5,61 | | 7,77 | | 10,76 | | -2,92 | | -1,88 | | -1,04 | | -3,39 | | 7,59 | 1,523 | 3,67 | 1,303 | 2,06 | 2,115 | 10,45 | 0,833 |
| Enchondr12 | 1 | 11,21 | | 7,55 | | 10,14 | | 13,77 | | -1,72 | | 0,06 | | 1,33 | | -0,37 | | 3,29 | 1,523 | 0,96 | 1,303 | 0,4 | 2,115 | 1,29 | 0,833 |
| Enchondr13 | 1 | 11,98 | | 10,92 | | 10,68 | | 15,39 | | -0,95 | | 3,44 | | 1,87 | | 1,24 | | 1,93 | 1,523 | 0,09 | 1,303 | 0,27 | 2,115 | 0,42 | 0,833 |
| Enchondr14 | 1 | 10,18 | | 7,53 | | 9,54 | | 14,89 | | -2,74 | | 0,04 | | 0,73 | | 0,74 | | 6,7 | 1,523 | 0,97 | 1,303 | 0,6 | 2,115 | 0,6 | 0,833 |
| Enchondr15 | 1 | 10,76 | 12,17 | 5,35 | 7,2 | 7,91 | 5,72 | 13,77 | 10,59 | -1,42 | -0,75 | -1,85 | -0,29 | 2,19 | -3,09 | 3,18 | 3,55 | 2,67 | 1,68 | 3,61 | 1,22 | 0,22 | 8,52 | 0,11 | 0,09 |
| Enchondr16 | 1 | 11,73 | | 7,6 | | 10,39 | | 14,01 | | -1,2 | | 0,11 | | 1,58 | | -0,13 | | 2,29 | 1,523 | 0,93 | 1,303 | 0,33 | 2,115 | 1,1 | 0,833 |
| | | | | | | | | | | | | | | | | | | | | | | | | | |
| ChonsG101 | 2 | 10,98 | | 8,26 | | 10,03 | | 16,04 | | -1,25 | | 1,31 | | 3,56 | | 6,19 | | 2,38 | 1,456 | 0,4 | 1,709 | 0,08 | 1,374 | 0,01 | 2,744 |
| ChonsG102 | 2 | 11,97 | | 7,02 | | 10,11 | | 13,68 | | -0,27 | | 0,08 | | 3,64 | | 3,83 | | 1,2 | 1,456 | 0,95 | 1,709 | 0,08 | 1,374 | 0,07 | 2,744 |
| ChonsG103 | 2 | 10,99 | 13,7 | 8,46 | 10,1 | 7,28 | 8,62 | 11,8 | 12,75 | -2,71 | 1,47 | -1,64 | 3,16 | -1,35 | 2,15 | -0,95 | 2,9 | 6,54 | 0,36 | 3,12 | 0,11 | 2,54 | 0,22 | 1,93 | 0,13 |
| ChonsG104 | 2 | 9,57 | | 6,63 | | 9,16 | | 12,08 | | -2,66 | | -0,31 | | 2,69 | | 2,23 | | 6,33 | 1,456 | 1,24 | 1,709 | 0,16 | 1,374 | 0,21 | 2,744 |
| ChonsG105 | 2 | | 12,31 | | 7,41 | | 6,15 | | 8,81 | | 0,08 | | 0,47 | | -0,31 | | -1,04 | | 0,95 | | 0,72 | | 1,24 | | 2,05 |
| ChonsG106 | 2 | 8,33 | 10,88 | 3,84 | 4,82 | 9,61 | 5,94 | 13,64 | 7,42 | -2,56 | -1,35 | -0,98 | -2,12 | 3,67 | -0,52 | 6,22 | -2,43 | 5,88 | 2,55 | 1,97 | 4,34 | 0,08 | 1,44 | 0,01 | 5,38 |
| ChonsG107 | 2 | 11,6 | 13,34 | 7,39 | 7,8 | 7,22 | 4,82 | 12,64 | 9,16 | -1,75 | 1,11 | -0,41 | 0,86 | 2,41 | -1,65 | 3,49 | -0,69 | 3,35 | 0,46 | 1,32 | 0,55 | 0,19 | 3,14 | 0,09 | 1,62 |
| ChonsG108 | 2 | 12,59 | 13,26 | 6,2 | 7,59 | 6,66 | 5 | 9,12 | 6,46 | -0,67 | 1,03 | -1,4 | 0,65 | 1,65 | -1,46 | 2,66 | -3,39 | 1,59 | 0,49 | 2,63 | 0,64 | 0,32 | 2,76 | 0,16 | 10,49 |
| ChonsG109 | 2 | 9,09 | 9,98 | 6,48 | 4,74 | 9,57 | 8,07 | 14,72 | 13,56 | -0,89 | -2,25 | 1,74 | -2,2 | 1,51 | 1,6 | 1,16 | 3,71 | 1,85 | 4,77 | 0,3 | 4,59 | 0,35 | 0,33 | 0,45 | 0,08 |
| ChonsG110 | 2 | 10,35 | 12,05 | 5,81 | 6,82 | 8,52 | 6,54 | 12,51 | 11,73 | -1,71 | -0,18 | -1,02 | -0,12 | 1,98 | 0,07 | 0,77 | 1,88 | 3,27 | 1,13 | 2,02 | 1,09 | 0,25 | 0,95 | 0,59 | 0,27 |
| ChonsG111 | 2 | 9,56 | 12,33 | 4,18 | 6,24 | 7,83 | 6,6 | 11,16 | 8,9 | -2,77 | 0,1 | -2,06 | -0,7 | 1,23 | 0,13 | 2,26 | -0,95 | 6,83 | 0,94 | 4,18 | 1,63 | 0,43 | 0,91 | 0,21 | 1,93 |
| | | | | | | | | | | | | | | | | | | | | | | | | | |
| ChonsG201 | 3 | 10,81 | | 4,12 | | 9,85 | | 12,51 | | -1,67 | | -3,34 | | 3,18 | | 1,7 | | 3,19 | 2,913 | 10,09 | 1,169 | 0,11 | 2,034 | 0,31 | 1,516 |
| ChonsG202 | 3 | 10,37 | | 4,8 | | 8,14 | | 13,37 | | -2,12 | | -2,66 | | 1,47 | | 2,56 | | 4,34 | 2,913 | 6,3 | 1,169 | 0,36 | 2,034 | 0,17 | 1,516 |
| ChonsG203 | 3 | 10,57 | 15,74 | 6,29 | 8,56 | 8,21 | 7,52 | 13,87 | 10,64 | -5,17 | 3,26 | -2,27 | 1,11 | 0,69 | 0,85 | 3,23 | -0,16 | 36 | 0,1 | 4,82 | 0,46 | 0,62 | 0,55 | 0,11 | 1,12 |
| ChonsG204 | 3 | 9,72 | 14,11 | 9,9 | 7,36 | 10,32 | 8,49 | 14,57 | 13,13 | -4,39 | 1,63 | 2,54 | -0,09 | 1,83 | 1,82 | 1,44 | 2,33 | 21 | 0,32 | 0,17 | 1,07 | 0,28 | 0,28 | 0,37 | 0,2 |
| ChonsG205 | 3 | 12,97 | 12,72 | 8,39 | 8,23 | 8,41 | 7,29 | 11,91 | 11,22 | 0,25 | 0,24 | 0,16 | 0,78 | 1,12 | 0,62 | 0,69 | 0,42 | 0,84 | 0,85 | 0,89 | 0,58 | 0,46 | 0,65 | 0,62 | 0,75 |
| ChonsG206 | 3 | 9,05 | 8,56 | 5,38 | 6,07 | 8,21 | 3,39 | 12,83 | 9,09 | 0,49 | -3,92 | -0,69 | -1,38 | 4,83 | -3,28 | 3,74 | -1,71 | 0,71 | 15,14 | 1,62 | 2,6 | 0,04 | 9,73 | 0,07 | 3,27 |
| ChonsG207 | 3 | 10,31 | | 5,78 | | 8,36 | | 13,38 | | -2,17 | | -1,68 | | 1,69 | | 2,57 | | 4,51 | 2,913 | 3,19 | 1,169 | 0,31 | 2,034 | 0,17 | 1,516 |
| ChonsG208 | 3 | 8,76 | 11,89 | 8,61 | 6,77 | 11,78 | 6,68 | 14,53 | 9,04 | -3,12 | -0,6 | 1,85 | -0,69 | 5,1 | 0,01 | 5,49 | -1,76 | 8,71 | 1,51 | 0,28 | 1,61 | 0,03 | 0,99 | 0,02 | 3,39 |
| ChonsG209 | 3 | 10,71 | 12,3 | 5,8 | 7,85 | 10,32 | 6,88 | 14,23 | 12,28 | -1,6 | -0,18 | -2,06 | 0,4 | 3,44 | 0,21 | 1,96 | 1,47 | 3,02 | 1,13 | 4,16 | 0,76 | 0,09 | 0,87 | 0,26 | 0,36 |
| ChonsG210 | 3 | 10,53 | 12,06 | 5,81 | 7,32 | 6,86 | 6,44 | 11,46 | 10,2 | -1,54 | -0,42 | -1,51 | -0,14 | 0,42 | -0,23 | 1,26 | -0,6 | 2,9 | 1,34 | 2,84 | 1,1 | 0,75 | 1,17 | 0,42 | 1,52 |
| | | | | | | | | | | | | | | | | | | | | | | | | | |
| ChonsG301 | 4 | 7,64 | 9,99 | 2,09 | 6,47 | 8,71 | 4,68 | 17,06 | 8,36 | -2,35 | -2,61 | -4,38 | -0,97 | 4,03 | -1,43 | 8,7 | -1,82 | 5,1 | 6,12 | 20,75 | 1,96 | 0,06 | 2,7 | 0,002 | 3,54 |
| ChonsG302 | 4 | 7,39 | 10,25 | 4,09 | 5,6 | 6,99 | 5,3 | 12,07 | 9,65 | -2,85 | -2,35 | -1,51 | -1,83 | 1,69 | -0,81 | 2,42 | -0,53 | 7,22 | 5,11 | 2,84 | 3,56 | 0,31 | 1,76 | 0,19 | 1,44 |
| ChonsG303 | 4 | 9,44 | | 5,16 | | 8,26 | | 13,89 | | -3,16 | | -2,27 | | 2,15 | | 3,71 | | 8,95 | 2,44 | 4,83 | 1,45 | 0,23 | 1,28 | 0,08 | 1,386 |
| ChonsG304 | 4 | 8,33 | 14,15 | 5,84 | 9,13 | 8,13 | 7,55 | 12,06 | 11,61 | -5,82 | 1,56 | -3,29 | 1,69 | 0,58 | 1,44 | 0,45 | 1,43 | 56,59 | 0,34 | 9,8 | 0,31 | 0,67 | 0,37 | 0,73 | 0,37 |
| ChonsG305 | 4 | 11,78 | 14,65 | 7,75 | 8,41 | 8,38 | 6,13 | 11,72 | 10,04 | -2,88 | 2,06 | -0,66 | 0,97 | 2,25 | 0,02 | 1,69 | -0,14 | 7,34 | 0,24 | 1,58 | 0,51 | 0,21 | 0,99 | 0,31 | 1,1 |
| ChonsG306 | 4 | 13,78 | 13,95 | 8,11 | 7,57 | 9,83 | 6,9 | 13,67 | 11,24 | -0,18 | 1,36 | 0,54 | 0,13 | 2,92 | 0,79 | 2,42 | 1,06 | 1,13 | 0,39 | 0,69 | 0,91 | 0,13 | 0,58 | 0,19 | 0,48 |
