# Supplementary material for: MiRNA-based expression signatures in differential diagnosis of enchondroma and chondrosarcoma
Source: J Bone Oncol. 2026 Apr 8;58:100761. doi: 10.1016/j.jbo.2026.100761 (PMC13141744; doi:10.1016/j.jbo.2026.100761)
Supplement: Supplementary Data 3 [file mmc3.pptx]

## Slide 1
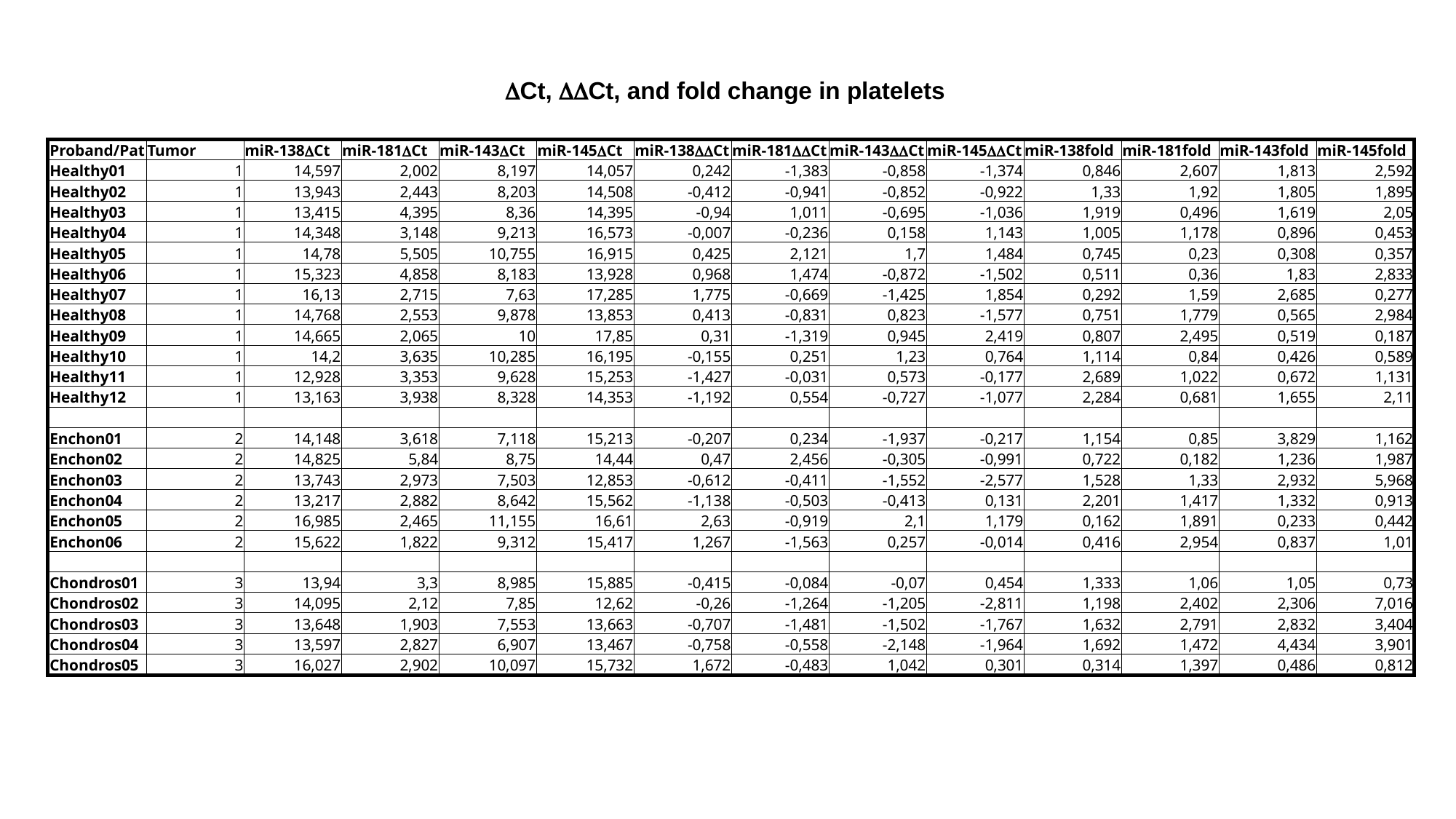

DCt, DDCt, and fold change in platelets
| Proband/Pat | Tumor | miR-138DCt | miR-181DCt | miR-143DCt | miR-145DCt | miR-138DDCt | miR-181DDCt | miR-143DDCt | miR-145DDCt | miR-138fold | miR-181fold | miR-143fold | miR-145fold |
| --- | --- | --- | --- | --- | --- | --- | --- | --- | --- | --- | --- | --- | --- |
| Healthy01 | 1 | 14,597 | 2,002 | 8,197 | 14,057 | 0,242 | -1,383 | -0,858 | -1,374 | 0,846 | 2,607 | 1,813 | 2,592 |
| Healthy02 | 1 | 13,943 | 2,443 | 8,203 | 14,508 | -0,412 | -0,941 | -0,852 | -0,922 | 1,33 | 1,92 | 1,805 | 1,895 |
| Healthy03 | 1 | 13,415 | 4,395 | 8,36 | 14,395 | -0,94 | 1,011 | -0,695 | -1,036 | 1,919 | 0,496 | 1,619 | 2,05 |
| Healthy04 | 1 | 14,348 | 3,148 | 9,213 | 16,573 | -0,007 | -0,236 | 0,158 | 1,143 | 1,005 | 1,178 | 0,896 | 0,453 |
| Healthy05 | 1 | 14,78 | 5,505 | 10,755 | 16,915 | 0,425 | 2,121 | 1,7 | 1,484 | 0,745 | 0,23 | 0,308 | 0,357 |
| Healthy06 | 1 | 15,323 | 4,858 | 8,183 | 13,928 | 0,968 | 1,474 | -0,872 | -1,502 | 0,511 | 0,36 | 1,83 | 2,833 |
| Healthy07 | 1 | 16,13 | 2,715 | 7,63 | 17,285 | 1,775 | -0,669 | -1,425 | 1,854 | 0,292 | 1,59 | 2,685 | 0,277 |
| Healthy08 | 1 | 14,768 | 2,553 | 9,878 | 13,853 | 0,413 | -0,831 | 0,823 | -1,577 | 0,751 | 1,779 | 0,565 | 2,984 |
| Healthy09 | 1 | 14,665 | 2,065 | 10 | 17,85 | 0,31 | -1,319 | 0,945 | 2,419 | 0,807 | 2,495 | 0,519 | 0,187 |
| Healthy10 | 1 | 14,2 | 3,635 | 10,285 | 16,195 | -0,155 | 0,251 | 1,23 | 0,764 | 1,114 | 0,84 | 0,426 | 0,589 |
| Healthy11 | 1 | 12,928 | 3,353 | 9,628 | 15,253 | -1,427 | -0,031 | 0,573 | -0,177 | 2,689 | 1,022 | 0,672 | 1,131 |
| Healthy12 | 1 | 13,163 | 3,938 | 8,328 | 14,353 | -1,192 | 0,554 | -0,727 | -1,077 | 2,284 | 0,681 | 1,655 | 2,11 |
| | | | | | | | | | | | | | |
| Enchon01 | 2 | 14,148 | 3,618 | 7,118 | 15,213 | -0,207 | 0,234 | -1,937 | -0,217 | 1,154 | 0,85 | 3,829 | 1,162 |
| Enchon02 | 2 | 14,825 | 5,84 | 8,75 | 14,44 | 0,47 | 2,456 | -0,305 | -0,991 | 0,722 | 0,182 | 1,236 | 1,987 |
| Enchon03 | 2 | 13,743 | 2,973 | 7,503 | 12,853 | -0,612 | -0,411 | -1,552 | -2,577 | 1,528 | 1,33 | 2,932 | 5,968 |
| Enchon04 | 2 | 13,217 | 2,882 | 8,642 | 15,562 | -1,138 | -0,503 | -0,413 | 0,131 | 2,201 | 1,417 | 1,332 | 0,913 |
| Enchon05 | 2 | 16,985 | 2,465 | 11,155 | 16,61 | 2,63 | -0,919 | 2,1 | 1,179 | 0,162 | 1,891 | 0,233 | 0,442 |
| Enchon06 | 2 | 15,622 | 1,822 | 9,312 | 15,417 | 1,267 | -1,563 | 0,257 | -0,014 | 0,416 | 2,954 | 0,837 | 1,01 |
| | | | | | | | | | | | | | |
| Chondros01 | 3 | 13,94 | 3,3 | 8,985 | 15,885 | -0,415 | -0,084 | -0,07 | 0,454 | 1,333 | 1,06 | 1,05 | 0,73 |
| Chondros02 | 3 | 14,095 | 2,12 | 7,85 | 12,62 | -0,26 | -1,264 | -1,205 | -2,811 | 1,198 | 2,402 | 2,306 | 7,016 |
| Chondros03 | 3 | 13,648 | 1,903 | 7,553 | 13,663 | -0,707 | -1,481 | -1,502 | -1,767 | 1,632 | 2,791 | 2,832 | 3,404 |
| Chondros04 | 3 | 13,597 | 2,827 | 6,907 | 13,467 | -0,758 | -0,558 | -2,148 | -1,964 | 1,692 | 1,472 | 4,434 | 3,901 |
| Chondros05 | 3 | 16,027 | 2,902 | 10,097 | 15,732 | 1,672 | -0,483 | 1,042 | 0,301 | 0,314 | 1,397 | 0,486 | 0,812 |
