# Supplementary material for: MiRNA-based expression signatures in differential diagnosis of enchondroma and chondrosarcoma
Source: J Bone Oncol. 2026 Apr 8;58:100761. doi: 10.1016/j.jbo.2026.100761 (PMC13141744; doi:10.1016/j.jbo.2026.100761)
Supplement: Supplementary Data 4 [file mmc4.pptx]

## Slide 1
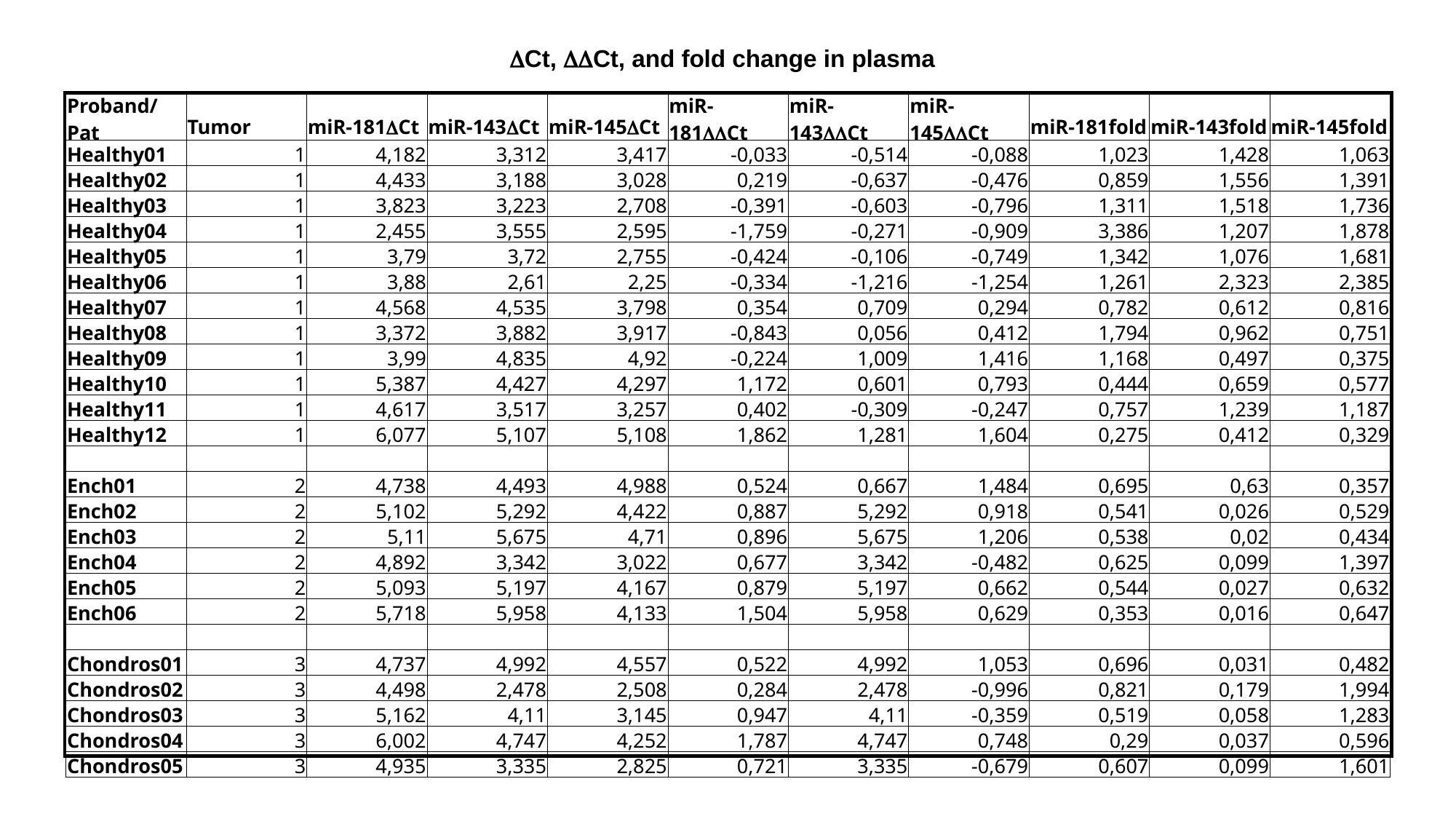

DCt, DDCt, and fold change in plasma
| Proband/Pat | Tumor | miR-181DCt | miR-143DCt | miR-145DCt | miR-181DDCt | miR-143DDCt | miR-145DDCt | miR-181fold | miR-143fold | miR-145fold |
| --- | --- | --- | --- | --- | --- | --- | --- | --- | --- | --- |
| Healthy01 | 1 | 4,182 | 3,312 | 3,417 | -0,033 | -0,514 | -0,088 | 1,023 | 1,428 | 1,063 |
| Healthy02 | 1 | 4,433 | 3,188 | 3,028 | 0,219 | -0,637 | -0,476 | 0,859 | 1,556 | 1,391 |
| Healthy03 | 1 | 3,823 | 3,223 | 2,708 | -0,391 | -0,603 | -0,796 | 1,311 | 1,518 | 1,736 |
| Healthy04 | 1 | 2,455 | 3,555 | 2,595 | -1,759 | -0,271 | -0,909 | 3,386 | 1,207 | 1,878 |
| Healthy05 | 1 | 3,79 | 3,72 | 2,755 | -0,424 | -0,106 | -0,749 | 1,342 | 1,076 | 1,681 |
| Healthy06 | 1 | 3,88 | 2,61 | 2,25 | -0,334 | -1,216 | -1,254 | 1,261 | 2,323 | 2,385 |
| Healthy07 | 1 | 4,568 | 4,535 | 3,798 | 0,354 | 0,709 | 0,294 | 0,782 | 0,612 | 0,816 |
| Healthy08 | 1 | 3,372 | 3,882 | 3,917 | -0,843 | 0,056 | 0,412 | 1,794 | 0,962 | 0,751 |
| Healthy09 | 1 | 3,99 | 4,835 | 4,92 | -0,224 | 1,009 | 1,416 | 1,168 | 0,497 | 0,375 |
| Healthy10 | 1 | 5,387 | 4,427 | 4,297 | 1,172 | 0,601 | 0,793 | 0,444 | 0,659 | 0,577 |
| Healthy11 | 1 | 4,617 | 3,517 | 3,257 | 0,402 | -0,309 | -0,247 | 0,757 | 1,239 | 1,187 |
| Healthy12 | 1 | 6,077 | 5,107 | 5,108 | 1,862 | 1,281 | 1,604 | 0,275 | 0,412 | 0,329 |
| | | | | | | | | | | |
| Ench01 | 2 | 4,738 | 4,493 | 4,988 | 0,524 | 0,667 | 1,484 | 0,695 | 0,63 | 0,357 |
| Ench02 | 2 | 5,102 | 5,292 | 4,422 | 0,887 | 5,292 | 0,918 | 0,541 | 0,026 | 0,529 |
| Ench03 | 2 | 5,11 | 5,675 | 4,71 | 0,896 | 5,675 | 1,206 | 0,538 | 0,02 | 0,434 |
| Ench04 | 2 | 4,892 | 3,342 | 3,022 | 0,677 | 3,342 | -0,482 | 0,625 | 0,099 | 1,397 |
| Ench05 | 2 | 5,093 | 5,197 | 4,167 | 0,879 | 5,197 | 0,662 | 0,544 | 0,027 | 0,632 |
| Ench06 | 2 | 5,718 | 5,958 | 4,133 | 1,504 | 5,958 | 0,629 | 0,353 | 0,016 | 0,647 |
| | | | | | | | | | | |
| Chondros01 | 3 | 4,737 | 4,992 | 4,557 | 0,522 | 4,992 | 1,053 | 0,696 | 0,031 | 0,482 |
| Chondros02 | 3 | 4,498 | 2,478 | 2,508 | 0,284 | 2,478 | -0,996 | 0,821 | 0,179 | 1,994 |
| Chondros03 | 3 | 5,162 | 4,11 | 3,145 | 0,947 | 4,11 | -0,359 | 0,519 | 0,058 | 1,283 |
| Chondros04 | 3 | 6,002 | 4,747 | 4,252 | 1,787 | 4,747 | 0,748 | 0,29 | 0,037 | 0,596 |
| Chondros05 | 3 | 4,935 | 3,335 | 2,825 | 0,721 | 3,335 | -0,679 | 0,607 | 0,099 | 1,601 |
